# LI-RADS-based hepatocellular carcinoma risk mapping using contrast-enhanced MRI and self-configuring deep learning

**DOI:** 10.1186/s40644-025-00844-6

**Published:** 2025-03-17

**Authors:** Róbert Stollmayer, Selda Güven, Christian Marcel Heidt, Kai Schlamp, Pál Novák Kaposi, Oyunbileg von Stackelberg, Hans-Ulrich Kauczor, Miriam Klauss, Philipp Mayer

**Affiliations:** 1 Clinic for Diagnostic and Interventional Radiology (DIR), Heidelberg University Hospital, Heidelberg, Germany; 2 Department of Radiology, Medical Imaging Centre, Semmelweis University, Budapest, Hungary; 3 Department of Radiology, Diskapi Yildirim Beyazit Training and Research Hospital, University of Health Sciences, Ankara, Turkey; 4 Department of Diagnostic and Interventional Radiology With Nuclear Medicine, Thoraxklinik at University of Heidelberg, Heidelberg, Germany; 5 Liver Cancer Center Heidelberg (LCCH), Heidelberg University Hospital, Heidelberg, Germany

**Keywords:** Hepatocellular carcinoma, Multiparametric MRI, Deep learning, Clinical guidelines

## Abstract

**Background:**

Hepatocellular carcinoma (HCC) is often diagnosed using gadoxetate disodium-enhanced magnetic resonance imaging (EOB-MRI). Standardized reporting according to the Liver Imaging Reporting and Data System (LI-RADS) can improve Gd-MRI interpretation but is rather complex and time-consuming. These limitations could potentially be alleviated using recent deep learning-based segmentation and classification methods such as nnU-Net. The study aims to create and evaluate an automatic segmentation model for HCC risk assessment, according to LI-RADS v2018 using nnU-Net.

**Methods:**

For this single-center retrospective study, 602 patients at risk for HCC were included, who had dynamic EOB-MRI examinations between 05/2005 and 09/2022, containing ≥ LR-3 lesion(s). Manual lesion segmentations in semantic segmentation masks as LR-3, LR-4, LR-5 or LR-M served as ground truth. A set of U-Net models with 14 input channels was trained using the nnU-Net framework for automatic segmentation. Lesion detection, LI-RADS classification, and instance segmentation metrics were calculated by post-processing the semantic segmentation outputs of the final model ensemble. For the external evaluation, a modified version of the LiverHccSeg dataset was used.

**Results:**

The final training/internal test/external test cohorts included 383/219/16 patients. In the three cohorts, LI-RADS lesions (≥ LR-3 and LR-M) ≥ 10 mm were detected with sensitivities of 0.41–0.85/0.40–0.90/0.83 (LR-5: 0.85/0.90/0.83) and positive predictive values of 0.70–0.94/0.67–0.88/0.90 (LR-5: 0.94/0.88/0.90). F1 scores for LI-RADS classification of detected lesions ranged between 0.48–0.69/0.47–0.74/0.84 (LR-5: 0.69/0.74/0.84). Median per lesion Sørensen–Dice coefficients were between 0.61–0.74/0.52–0.77/0.84 (LR-5: 0.74/0.77/0.84).

**Conclusion:**

Deep learning-based HCC risk assessment according to LI-RADS can be implemented as automatically generated tumor risk maps using out-of-the-box image segmentation tools with high detection performance for LR-5 lesions. Before translation into clinical practice, further improvements in automatic LI-RADS classification, for example through large multi-center studies, would be desirable.

**Supplementary Information:**

The online version contains supplementary material available at 10.1186/s40644-025-00844-6.

## Background

Hepatocellular carcinoma (HCC) is a leading cause of cancer-related death worldwide and early detection is pivotal. According to recent guidelines [1–4], the characteristic appearance of HCC with radiological imaging is sufficient for its diagnosis without the need for biopsy in patients who are at high risk for HCC and when there is curative intent. The characteristic vascular pattern of HCC with marked enhancement in the arterial phase and washout appearance in the later phases can be observed in dynamic contrast-enhanced (DCE) imaging studies, among which magnetic resonance imaging (MRI) has the highest sensitivity and specificity [5]. The use of hepatocyte-specific agents, such as gadoxetate disodium, further increases the per-lesion sensitivity of DCE-MRI, particularly for small HCCs [5], and may be useful in the prediction of histopathological features such as microvascular invasion [6].

Non-standardized imaging protocols, image interpretation, and reporting can lead to inadequate assessment of liver lesions and inaccurate communication of HCC risk [7]. To reduce inconsistencies standardized guidelines have been proposed [8]. The most popular system is the Liver Imaging Reporting And Data System (LI-RADS) [8].

Although standardized HCC imaging and diagnostics according to LI-RADS is now widely implemented in academic centers [8], its adoption by non-academic radiologists is lagging, partly due to its complexity [9]. Novice users and users in high-volume private practice may struggle with its use [10].

Deep learning (DL) methods could provide a solution in the form of automated tools for lesion detection, segmentation, and characterization [11]. Out-of-the-box DL frameworks, such as nnU-Net, lift model development workload, while providing state-of-the-art segmentation results [12]. This enables further development of tools for automated segmentation of anatomical structures and pathologies [13], which can be used for HCC diagnostics [11]. However, most previous segmentation-based studies have either only evaluated histologically confirmed HCC cases or proposed complex multistep pipelines which hinders the utility of these methods.

The current study aims to evaluate a simple, yet realistic approach, where the available scans of DCE-MRI examinations are automatically converted via nnU-Net into tumor risk maps, which can be used as an assistance tool for reporting, disease burden quantification, large-scale data annotation, and analysis, or as an always-available standardized reference of reporting quality in the clinical routine.

## Methods

### Patients

This study was approved by the Institutional Review Board and was performed in accordance with the 1964 Helsinki Declaration and its later amendments, informed consent was waived.

**Fig. 1:**
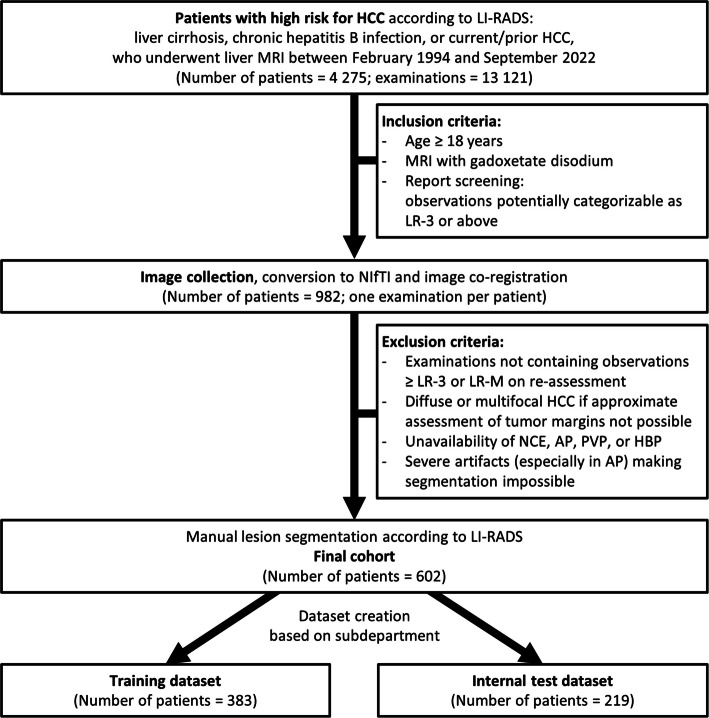
Flowchart of study participants, as well as inclusion and exclusion criteria of the study. HCC – hepatocellular carcinoma; LI-RADS – Liver Imaging Reporting and Data System; MRI – magnetic resonance imaging, NIfTI – Neuroimaging Informatics Technology Initiative file format; NCE – pre-contrast T1-weighted image; AP – late arterial phase; PVP – portal venous phase; HBP – hepatobiliary phase T1-weighted image; LR-3 and LR-M – LI-RADS categories

In the current retrospective single-center study, patients were identified via semi-automatic report search and filtering within the clinics Radiology Information System. The search included MRI examinations performed on patients at risk of developing HCC (reports mentioning cirrhosis, hepatitis B infection, or current or prior HCC) between February 1994 and September 2022 (Fig. 1). The resulting examinations were filtered further to include DCE-MRI examinations performed with gadoxetate disodium (EOB-MRI, Primovist®, Bayer Vital GmBH). Of these filtered examinations, one examination per patient (≥ 18 years old) containing the highest number of lesions, potentially categorizable as LR-3 or above according to LI-RADS v2018, was included. Patient exclusion criteria were cirrhosis due to congenital hepatic fibrosis or cirrhosis due to vascular causes.

MRI exclusion criteria were examinations not containing lesions ≥ LR-3 or LR-M; diffuse or multifocal HCC if approximate assessment of tumor margins not possible; unavailability of any of late arterial (AP), portal venous (PVP), hepatobiliary (HBP), or pre-contrast T1-weighted (NCE) phase; severe artifacts on AP. Cases with missing or noisy images of any other MRI sequence type described in the LI-RADS imaging protocol were not excluded.

### MRI examinations

MRI examinations were performed using five different MRI scanners. Examinations used for training were acquired using two scanners. The patients in the test cohort were scanned with three different scanners (Table 1). MRI parameters are listed in Table 2.

**Table 1:** Patient, scanner, and lesion characteristics (all segmented areas) in the two cohorts

	Training dataset (*n* = 383)	Internal test dataset (*n* = 219)
Patient characteristics		
Age (years)^a^	62.0 (32–90)	61.0 (28–84)
- Male^b^	300 (78.3)	173 (79.0)
- Female^b^	83 (21.7)	46 (21.0)
Etiology^b^		
- Alcohol	130 (33.9)	83 (37.9)
- HCV	129 (33.7)	55 (25.1)
- HBV	57 (14.9)	29 (13.2)
- NASH	17 (4.4)	14 (6.4)
- Other	72 (18.8)	37 (16.9)
Cirrhosis^b^		
Yes		
- CHILD-A	220 (57.4)	139 (63.5)
- CHILD-B	105 (27.4)	49 (22.4)
- CHILD-C	41 (10.7)	11 (5.0)
- NA	3 (0.8)	1 (0.5)
No		
- Pathology-proven HCC	12 (3.1)	18 (8.2)
- Chronic HBV	2 (0.2)	1 (0.5)
MRI characteristics^b^		
MRI model (field strength in Tesla)		
- Siemens Avanto^e^ (1.5)	288 (75.2)	-
- Siemens Avanto fit^e^ (1.5)	95 (24.8)	-
- Siemens Aera^e^ (1.5)	-	186 (84.9)
- Siemens Symphony^e^ (1.5)	-	17 (7.8)
- Magnetom Vida^e^ (3.0)	-	16 (7.3)
Lesion characteristics		
Lesion diameter (mm)^c^		
- LR-3	16 (12, 19)	15 (12, 20)
- LR-4	17 (13, 25)	18 (14, 25)
- LR-5	28 (19, 40)	34 (23, 55)
- LR-M	24 (17, 40)	32 (21, 58)
All	19 (14, 27)	20 (14, 30)
Lesion count^d^		
- LR-3	661 (39.9)	304 (34.8)
- LR-4	444 (26.8)	309 (35.4)
- LR-5	416 (25.1)	220 (25.2)
- LR-M	136 (8.2)	41 (4.7)
All	1657	874

^a^Median (minimum–maximum range), ^b^absolute number (percentage of all patients), ^c^median (lower, upper quartile), ^d^absolute number (percentage of lesions), ^e^Product of Siemens Healthineers. *CHILD-A, -B, -C* Child-Turcott-Pugh scores, *HBV* hepatitis B virus, *HCV* hepatitis C virus, *HCC* hepatocellular carcinoma, *LR-3, -4, -5, -M* Liver Imaging Reporting and Data System categories, *mm* millimeter, *MRI* magnetic resonance imaging, *n* number of patients, *NASH* non-alcoholic steatohepatitis

**Table 2:** Magnetic resonance imaging parameters of the two cohorts per U-Net input channel

Input channel	DS	% avail	TE (ms)	TR (ms)	FA (°)	PS (mm)	ST (mm)	b-value (s/mm^2^)
NCE	Tr	100	1.44; 0.92—4.77	3.5; 2.64—7.09	12; 10—15	1.07; 0.48—1.41	3; 3—3.7	
	Ts	100	2.16; 1.25—3.33	4.69; 3.57—6.66	10; 9—25	1.25; 0.68—1.76	3; 2.77—3.5	
AP	Tr	100	1.44; 0.92—4.77	3.5; 2.64—7.09	12; 10—15	1.07; 0.48—1.41	3; 3—3.7	
	Ts	100	2.16; 1.25—2.28	4.69; 3.57—5.4	10; 9—25	1.25; 0.68—1.76	3; 2.77—3.5	
PVP	Tr	100	1.44; 0.92—4.77	3.5; 2.64—7.09	12; 10—15	1.07; 0.48—1.41	3; 3—3.7	
	Ts	100	2.16; 1.25—2.28	4.69; 3.57—5.4	10; 9—25	1.25; 0.68—1.76	3; 2.77—3.5	
TRA	Tr	90.6	1.13; 0.98 – 6	3.03; 2.51—158	12; 10—70	1.25; 0.86—1.95	3; 2.5—6.5	
	Ts	99.1	2.16; 1.22—2.28	4.69; 3.41—5.4	10; 9—25	1.25; 0.68—1.76	3; 2—3.5	
HBP	Tr	100	1.45; 0.95—2.39	3.5; 2.47—6.81	12; 10—40	1.09; 0.47—1.47	3; 3—3.7	
	Ts	100	2.16; 1.25—2.39	4.69; 3.57—6.66	10; 9—40	1.25; 0.68—1.76	3; 2.77—3.5	
IP	Tr	49.3	4.77; 4.76—4.78	7.59; 6.64—173	25; 10—70	1.04; 0.7—1.38	3; 3—6	
	Ts	71.2	4.76; 4.76—4.78	100; 6.71—115	70; 10—70	0.62; 0.53—1.3	6; 3—6	
OOP	Tr	49.1	2.39; 2.38—2.39	7.59; 6.64—173	25; 10—70	1.04; 0.7—1.38	3.1; 3—6	
	Ts	71.2	2.38; 2.27—2.39	100; 6.71—115	70; 10—70	0.62; 0.53—1.3	6; 3—6	
T2H	Tr	99.7	68; 66 – 402	801; 600—1600	160; 99—180	1.09; 0.91—1.95	6; 4—6	
	Ts	95.4	134; 81 – 137	680; 450—1400	159; 113—180	1.25; 0.59—1.56	6; 4—6	
T2B	Tr	96.6	101; 1.59 – 116	4353; 465 – 13,646	140; 56—160	1.19; 0.67—1.95	6; 3—7	
	Ts	71.2	84; 79 – 109	5573; 1330—19,758	150; 101—180	1.22; 0.89—1.88	6; 5—6	
T2LTE	Tr	49.9	251; 171 – 255	1000; 1000—3490	150; 150—180	1.25; 1.12—1.68	6	
	Ts	70.3	162; 79—226	1800; 1800 – 7644	180; 131—180	1.48; 0.99—1.84	6	
DWI-L	Tr	82.5	73; 57—79	4600; 2500 – 15,742	90	1.98; 1.42—2.6	5; 4—7	50
	Ts	91.8	60; 54—78	5400; 3500 – 8600	90	1.98; 1.34—3.38	6; 5—6	50; 50—100;
DWI-M	Tr	59.5	73; 62—131	2500; 2500 – 7731	90	1.98; 1.43—2.6	5; 4—7	300; 300—500;
	Ts	65.3	60; 54—78	5400; 2200 – 8600	90	2.08; 1.34—3.23	6; 5—6	400; 300—500;
DWI-H	Tr	84.9	73; 57—131	4600; 2500 – 7731	90	1.98; 1.42—2.6	5; 4—7	600; 600—1000;
	Ts	91.8	60; 54—78	5400; 2800 – 8600	90	1.98; 1.34—3.38	6; 5—6	800; 600—900;
ADC	Tr	83.6				1.98; 1.42—2.6	5; 4—7	
	Ts	91.8				1.98; 1.34—3.38	6; 5—6	

% avail. refers to the percentage of cases where the given image type was available. Where multiple values are presented, the first value is the median and the second pair of values is the range. MRI parameter values with two or more digits are rounded to integers. Input channels: NCE—non-contrast T1; AP—arterial phase; PVP—portal venous phase; TRA—transitional phase; HBP—hepatobiliary phase; IP—in-phase; OOP—out-of-phase; T2H—T2-weighted HASTE; T2B—T2-weighted BLADE; T2LTE—Multiple types of T2-weighted sequences with longer time to echo; DWI-L, DWI-M, DWI-H—diffusion-weighted imaging with three increasing B-value ranges; ADC—apparent diffusion coefficient. *DS* dataset, *FA* flip angle, *PS* pixel spacing, *ST* slice thickness, *TE* time to echo, *TR* time to repetition, *Tr* training dataset, *Ts* internal test dataset, *mm* millimeter, *ms* millisecond, *s* second

### Manual image segmentation

The filtered examinations were pseudonymized and exported from the Pictures Archiving and Communication System via ADIT (https://github.com/openradx/adit). Exported examinations were converted to NIfTI format, and co-registered to NCE scans using the 3D Slicer Elastix module [14]. 3D Slicer v5.1.0 [15] was used for manual image segmentation. Manual segmentation was performed by a radiology trainee (514 cases) with 3 years of experience in liver MRI analysis and a board-certified junior radiologist (88 cases) with 5 years of experience in abdominal imaging. Segmentations were proofread by a board-certified radiologist with 11 years of experience in abdominal imaging. All clinical information was available to the observers.

Segmentation was performed based on the co-registered images for each examination by marking lesions in a single semantic segmentation mask according to LI-RADS v2018. Lesions were manually classified as LR-3, LR-4, LR-5, or LR-M based on the co-registered contrast-enhanced scans by also considering ancillary LI-RADS features in all MRI scans. Subtraction images were available for lesion classification as they were reported to improve detection of AP hyperenhancement in EOB-MRI [16]. The major feature threshold growth was not used for categorization. Contrary to the original LI-RADS recommendations, pathologically proven tumors were also classified solely according to their MRI appearance. Observers were instructed to perform the manual segmentation of the lesions on the NCE or any of the DCE images, (while also taking into account lesion appearance on other MRI sequences) depending on which phase showed the clearest and most accurate lesion margins and least anatomic distortion [17]. As recommended in the LI-RADS manual (for size measurements), AP images were only used for segmentation if the lesion margins were not clearly visible on any other phase to avoid size overestimation due to corona enhancement or perilesional enhancement [17]. Lesions with enhancing capsules were commonly segmented on the portal venous phase which was reported to be the most accurate phase for detection of a capsule in EOB-MRI [18]. LR-3 were often segmented on the AP (non-rim AP hyperenhancement is the only major LI-RADS feature with prevalence ≥ 50% in LR-3 lesions) or HPB phase (HPB hypointensity occurs in ~ 20% of LR-3 lesions) [19].

A publicly available liver segmentation model [20] was used to create whole liver segmentations on the co-registered AP images to identify erroneous segmentations of lesions outside the liver boundaries and improve segmentation quality. The segmentations from the publicly available model were used as ground truth for the liver class during training.

The etiology of the chronic liver disease (e.g. alcohol, chronic virus hepatitis) is known to influence liver size, shape, and texture [21]. To generate a widely applicable model, cases with different etiologies of the chronic liver disease were pooled.

### Model development

The final cohort was split into two datasets according to the corresponding subdepartment, where the scans were acquired. The larger dataset was used for training with nnU-Net, while the smaller dataset was used for internal testing. Images within each examination were split among 14 groups, each assigned to a U-Net input channel (Table 2). Missing images within one examination were replaced by images consisting of only zero values. Model creation, training, planning, and data preprocessing for training, such as augmentation were set by the nnU-Net pipeline without modification. Configurations along with learning curves are available as supplementary materials (Additional files 1–7).

### External validation

An external evaluation was performed on the LiverHccSeg dataset [22]. Delayed phase (DEL) images were used instead of transitional phase (TRA) and HBP images when an extracellular contrast agent was used. Tumors were re-categorized according to LI-RADS v2018 based on the available NIfTI files, and all DICOM series fitting to an input channel were included. Examinations with inadequate image quality were excluded.

### Statistical evaluation

**Fig. 2:**
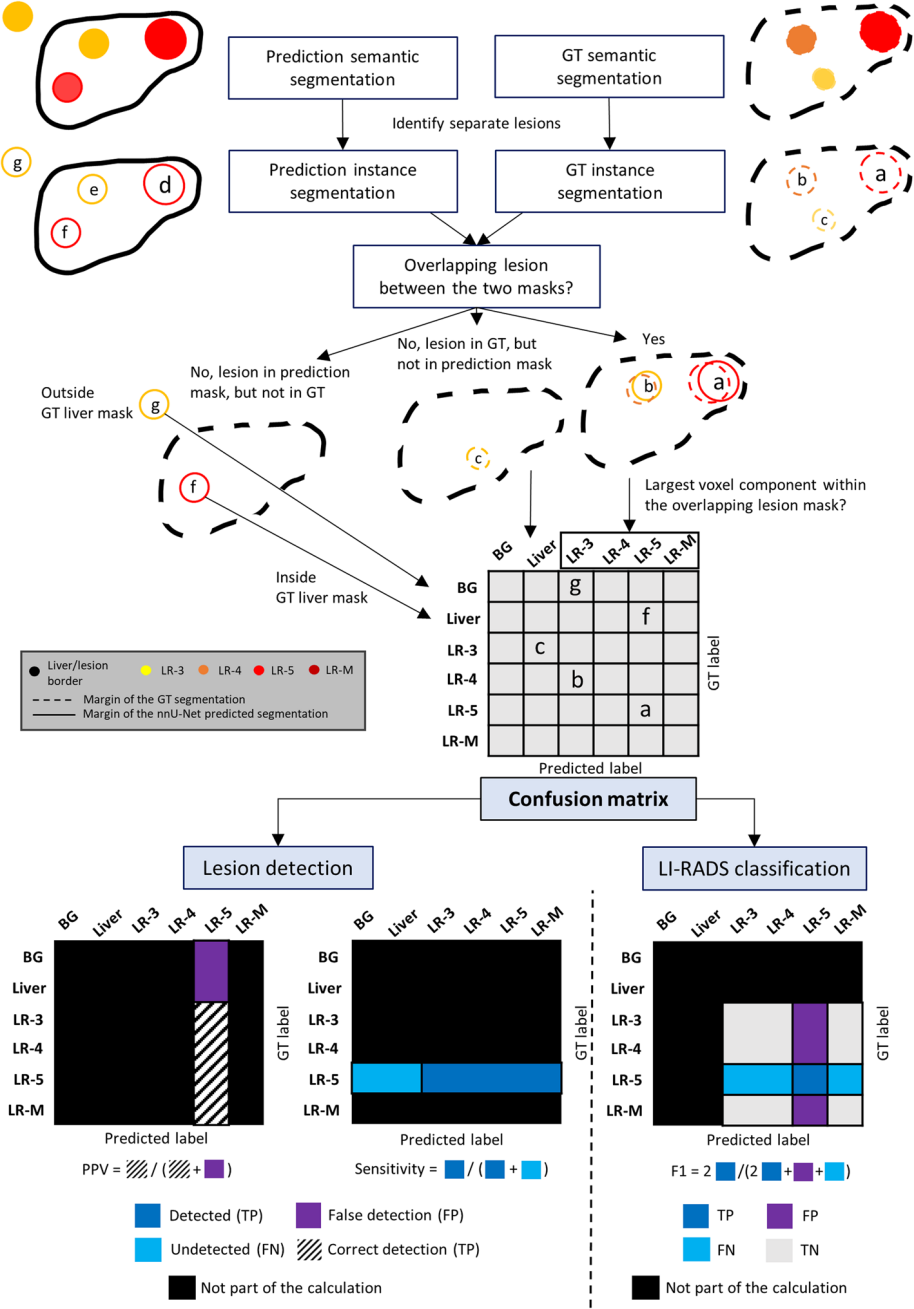
Flowchart illustrating the calculation of each evaluation metric. AP: arterial phase T1-weighted; BG: background; FN: false negative; FP: false positive; GT: ground truth; LR-3, -4, -5, -M: Liver Imaging Reporting and Data System categories; PPV: positive predictive value; TN: true negative; TP: true positive

To allow for a more accurate interpretation of the results, model performance was evaluated in segmentation (semantic and instance segmentation), lesion detection, and the LI-RADS classification of detected lesions. A simplified flowchart of how each of these tasks is evaluated based on the semantic segmentation masks that the U-Net creates is shown in Fig. 2.

#### Segmentation

Sørensen–Dice coefficients (DSC) and concordance correlation coefficients (CCC) were calculated to measure segmentation quality and volume agreement. DSC was calculated on an examination and lesion level. Examination level DSC measured the spatial overlap between predicted segmentations and ground truth segmentations in one examination, calculated for cases where a manually marked lesion was present in the given LI-RADS category. Lesion level DSC measured the spatial overlap between predicted segmentations and ground truth segmentations for one certain LI-RADS category of the ground truth lesions, not taking into account the LI-RADS category of the predicted lesions. DSCs are reported as median (lower, upper quartile), CCCs as CCC value (lower, upper bound of 95% confidence interval).

#### Confusion matrices

LI-RADS categories between ground truth lesions and predicted segmentations were automatically compared. The predicted LI-RADs category were determined using the following rules. If predicted segmentations with more than one LI-RADS category overlapped with one ground truth lesion, the predicted segmentation which showed the largest overlap with the ground truth lesion determined the predicted LI-RADS category. A predicted cluster of voxels that does not overlap with a ground truth segmentation was considered a false-positive finding. If this cluster contained different voxels with more than one LI-RADS category, the largest portion of voxels assigned to one category determined the predicted LI-RADS category for this false-positive finding.

Based on these results confusion matrices are created (Fig. 2).

#### Detection

Sensitivity in the context of lesion detection refers to the portion of ground truth lesions with a certain LI-RADS category that overlapped with predicted lesions with any LI-RADS category, compared to all ground truth lesions with this certain LI-RADS category.

Positive predictive value (PPV) in the context of lesion detection refers to the portion of predicted lesions with a certain LI-RADS category that overlapped with any ground truth lesion irrespective of the ground truth LI-RADS category.

#### Classification

Classification metrics are calculated for ground truth lesions that were segmented by nnU-Net (predicted lesions). Sensitivity, specificity, negative and positive predictive values (NPV), F1 score, and Cohen’s kappa values are derived from the created confusion matrices (Fig. 2) along with bootstrapped confidence intervals (lower, upper bound of 95% confidence interval).

To assess the contribution of each input channel, the same evaluation process is repeated for each input by replacing the respective image with an image containing only zero values.

All lesion level metrics are calculated for lesions ≥ 10 mm. For additional information see the supplementary materials (Additional file 1.docx) [23–25].

## Results

### Study population

Out of 4275 patients identified, 602 were included in the analysis. Included examinations were performed between May 2005 and September 2022. The flowchart of inclusion and exclusion steps is shown in Fig. 1.

**Fig. 3:**
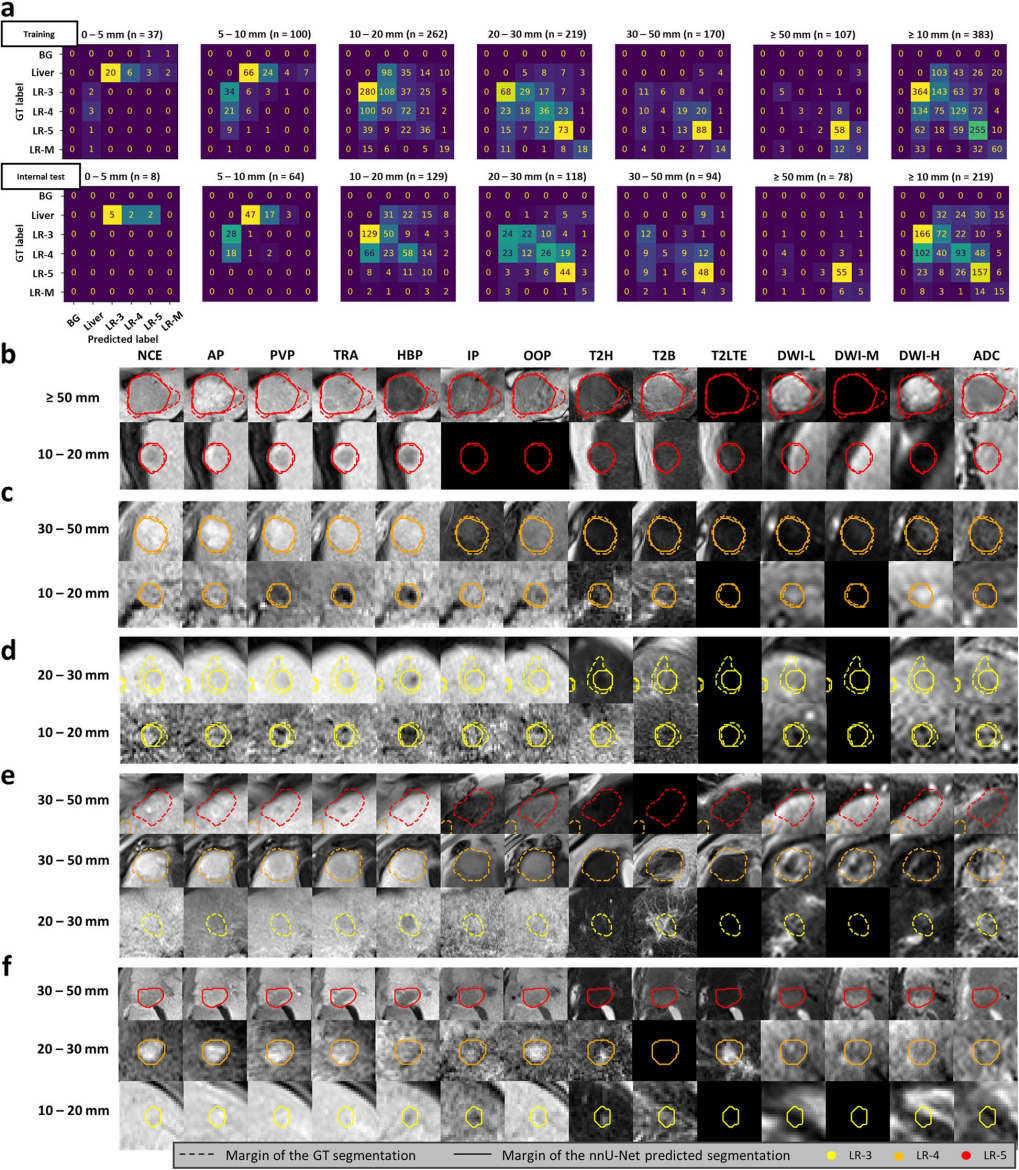
Confusion matrices from the two internal datasets with example lesions from the internal test dataset. **a** Confusion matrices comparing U-Net predicted and manually drawn ground truth lesions split up into subplots based on the largest axial diameter of the ground truth segmentation (or predicted segmentation for lesions not marked in the ground truth segmentation) lesions. **b-d** True positive (segmented in the correct category) examples. **e** False negative (undetected) examples. **f** False positive (manually not marked) examples. Numbers on the left in the given row indicate largest axial diameter range from which the lesion is sampled. Input channels in order: NCE – non-contrast-enhanced, pre-contrast T1; AP, PVP, TRA—arterial, portal venous, transitional phase contrast-enhanced T1; HBP – hepatobiliary phase contrast-enhanced T1; IP, OOP – in- and out-of-phase T1-weighted sequences; T2H – T2-weighted HASTE; T2B – T2-weighted BLADE; T2LTE – multiple types of T2-weighted images with longer time to echo; DWI-L, -M, -H – diffusion-weighted imaging with three increasing b-value ranges; ADC – apparent diffusion coefficient maps. BG: background; mm: millimeter; GT: ground truth: n: number of patients; LR-3, LR-4, LR-5, LR-M: LI-RADS categories

**Fig. 4:**
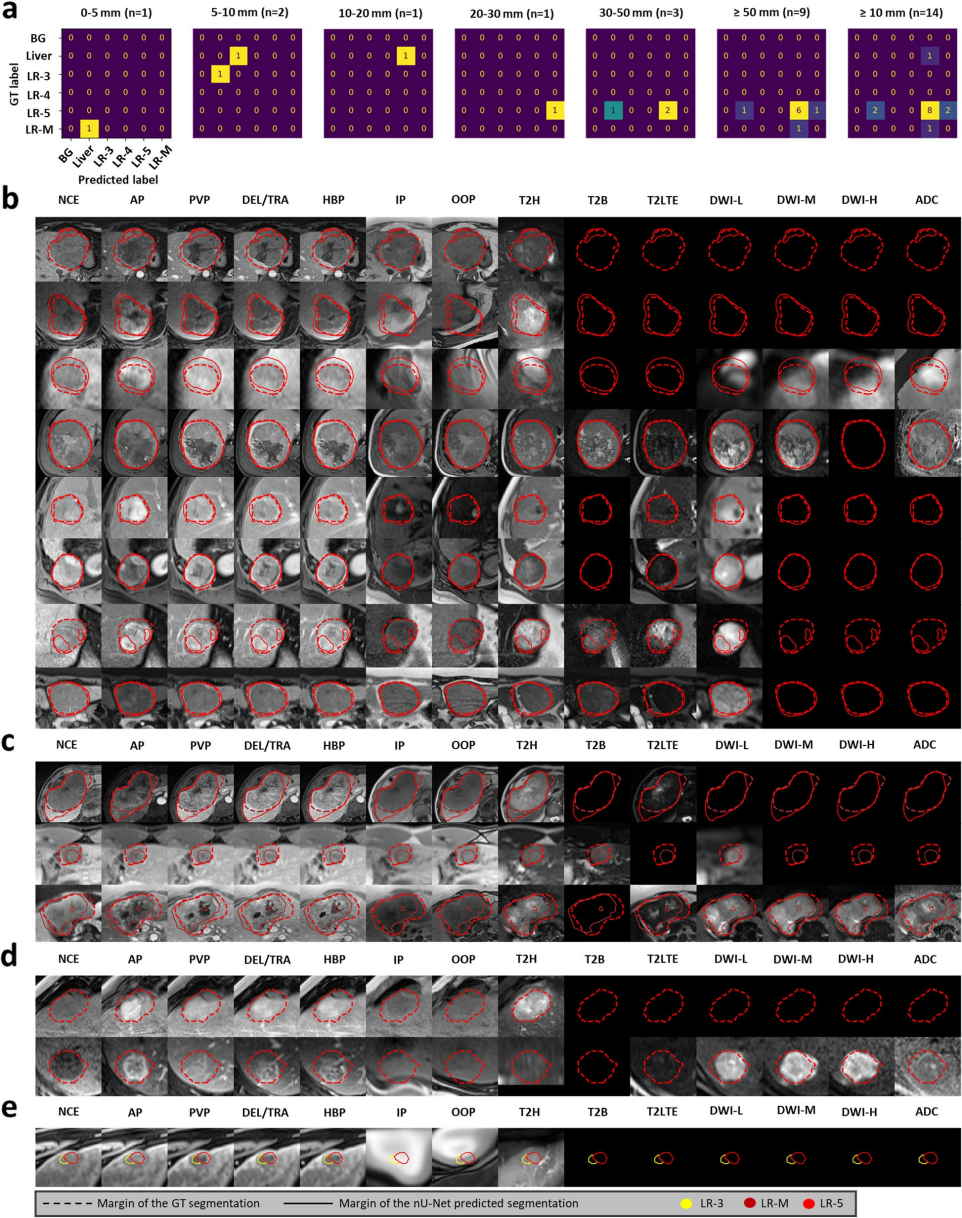
External test results. **a** Confusion matrices of classification results. **b**-**e** Input images per U-Net input channel overlapped with the ground truth segmentations and the nnU-Net segmentations (**b** true positives, **c** misclassified lesions, **d** not detected, **e** false positive detection). Input channels in order from left to right: NCE – non-contrast-enhanced, pre-contrast T1; AP, PVP, DEL/TRA—arterial, portal venous, delayed/transitional phase contrast-enhanced T1; HBP – hepatobiliary phase contrast-enhanced T1; IP, OOP – in- and out-of-phase T1-weighted sequences; T2H – T2-weighted HASTE; T2B – T2-weighted BLADE; T2LTE – multiple types of T2-weighted images with longer time to echo; DWI-L, -M, -H – diffusion-weighted imaging with three increasing b-value ranges; ADC – apparent diffusion coefficient maps. BG – background, LR-3, -4, -5, -M – included LI-RADS categories; mm – millimeter

Patient, scanner, and lesion characteristics are described in Table 1. 1657 and 874 marked areas were automatically identified from the manual semantic segmentations in the training and test datasets, of which 416 and 220 were marked as LR-5. Stratification of lesions based on their largest axial diameter is shown in Figs. 3 and 4. Most patients had less severe (CHILD-A) cirrhosis, while all Child–Pugh score categories were present in both groups, as well as patients without cirrhosis. The summary of MRI parameters of the scans used is available in Table 2.

### Semantic segmentation

For liver segmentation, in the internal test dataset, median DSC of 0.96 (0.92, 0.97) of the predicted segmentations compared to the segmentations from the public model and of 0.99 (0.98, 1.00) compared to the manually corrected outputs were calculated. In the training dataset, the median DSC between the predicted segmentations and the segmentations from the public model was 0.97 (0.95, 0.97).

For liver volume estimation, in the internal test dataset, a CCC of 0.73 (0.51, 0.85) was calculated between model predictions compared to liver segmentations acquired from the public model, and a CCC of 0.98 (0.96, 0.99) was achieved compared to manually corrected segmentations. In the training dataset, CCC for liver volume estimation was 0.85 (0.76, 0.90). The liver segmentations were not corrected manually due to the large number of cases in both datasets.

For liver lesion semantic segmentation, the highest overlap between ground truth and predicted segmentations was for LR-5 in the training and internal test cohorts (DSC_training_ = 0.72, DSC_test_ = 0.76) with CCC values of 0.86 and 0.94. In both cohorts, DSC and CCC values were markedly lower in the LR-3, LR-4, and LR-M categories (DSC ≤ 0.07, CCC ≤ 0.35).

**Fig. 5:**
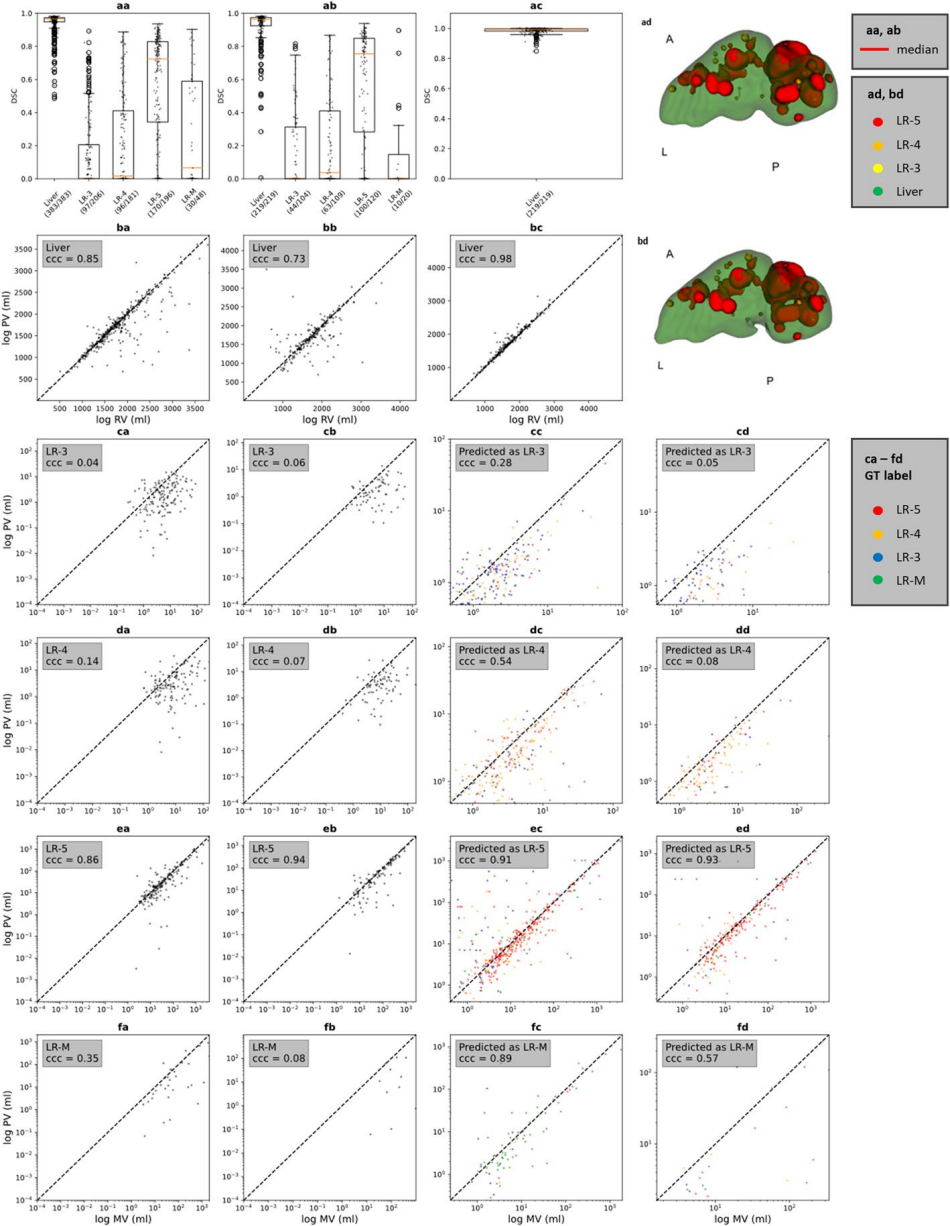
Evaluation of predicted segmentations. **aa**, **ab**: Sørensen–Dice coefficient (DSC) per LI-RADS category (category present in the ground truth segmentations) in the training (**aa**) and internal test (**ab**) cohorts. Box and scatter plot of DSCs of the test dataset liver segmentations (**ac**). Plots **ad** and **bd**: Internal test dataset output segmentation before (**ad**) and after manual correction (**bd**). Plots **aa**-**ac**: vertical axes show segmentation classes and the ratio of marked cases compared to all cases where the class was present. **ba**, **bb**: Liver volume calculated from predicted segmentations versus from ground truth segmentations for training (**ba**) and test (**bb**) datasets. **bc**: Liver volumes calculated from the segmentations of our model compared to volumes calculated from the manually corrected segmentations of our model in the test dataset. Plots **ca**-**fd**: compare the predicted segmentations from our model and manually drawn ground truth segmentations per LI-RADS category. Plots **ca**, **cb**, **da**, **db**, **ea**, **eb**, **fa**, **fb** compare whole segmentation volumes in the training (**ca**, **da**, **ea**, **fa**) and test (**cb**, **db**, **eb**, **fb**) datasets. Plots **cc**, **cd**, **dc**, **dd**, **ec**, **ed**, **fc**, **fd** compare lesion volumes of the manually marked ground truth lesions to the volume of any overlapping predicted lesion in the training (**cc**, **dc**, **ec**, **fc**) and test (**cd**, **dd**, **ed**, **fd**) datasets. CCC: concordance correlation coefficient; ml: milliliter; A, L, R, P (**ad**, **bd**): anterior, left, right, and posterior directions; MV: volumes calculated from manual segmentations; RV: reference volumes; PV: volumes calculated from the segmentations of our model

Segmentation and volumetry metrics are presented in detail in Table 3 and Fig. 5.

**Table 3:** Semantic and instance segmentation metrics

	**LR-3**	**LR-4**	**LR-5**	**LR-M**
**Semantic segmentation**				
**Mean DSC**^a^				
Tr	0.15 (0.23)	0.23 (0.29)	0.57 (0.32)	0.29 (0.33)
Ts	0.16 (0.24)	0.21 (0.27)	0.58 (0.34)	0.15 (0.27)
Ex	-	-	0.45 (0.40)	-
**Median DSC**^b^				
Tr	0.00 (0.00, 0.21)	0.02 (0.00, 0.41)	0.72 (0.34, 0.83)	0.07 (0.00, 0.59)
Ts	0.00 (0.00, 0.31)	0.04 (0.00, 0.41)	0.76 (0.28, 0.85)	0.00 (0.00, 0.15)
Ex	-	-	0.53 (0.00, 0.85)	-
**CCC**^c^				
Tr	0.04 (0.00, 0.13)	0.14 (0.06, 0.22)	0.86 (0.62, 0.97)	0.35 (0.11, 0.71)
Ts	0.06 (0.00, 0.16)	0.07 (0.02, 0.18)	0.94 (0.83, 0.97)	0.08 (0.00, 0.33)
Ex	-	-	0.53	-
**Instance segmentation** (lesions ≥ 10 mm)				
**Mean DSC**^a^				
Tr	0.56 (0.23)	0.61 (0.21)	0.63 (0.27)	0.59 (0.25)
Ts	0.53 (0.24)	0.59 (0.23)	0.66 (0.26)	0.45 (0.31)
Ex	-	-	0.68 (0.29)	-
**Median DSC**^b^				
Tr	0.61 (0.42, 0.73)	0.67 (0.52, 0.77)	0.74 (0.54, 0.82)	0.66 (0.44, 0.78)
Ts	0.58 (0.35, 0.74)	0.66 (0.46, 0.76)	0.77 (0.58, 0.84)	0.52 (0.10, 0.70)
Ex	-	-	0.84 (0.65, 0.87)	-
**CCC**^c^				
Tr	0.28 (0.08, 0.73)	0.54 (0.39, 0.73)	0.91 (0.78, 0.97)	0.89 (0.73, 0.97)
Ts	0.05 (0.01, 0.26)	0.08 (0.02, 0.64)	0.93 (0.80, 0.97)	0.57 (0.20, 0.95)
Ex	-	-	0.91	-

In parentheses: ^a^standard deviation, ^b^lower, upper quartile, ^c^lower, upper bound of 95% confidence interval. *CCC* concordance correlation coefficient, *DSC* Sørensen–Dice coefficient, *LR-3, LR-4, LR-5, LR-M* LI-RADS categories, *mm* millimeters, *Tr* training dataset, *Ts* internal test dataset, *Ex* external test dataset

### Instance segmentation

Lesions level median DSCs ranged between 0.61–0.74 in training and 0.52–0.77 in the internal test cohort. CCCs between the predicted and ground truth volume of lesions ranged between 0.28–0.91 for lesions detected in the training cohort, and accordingly 0.05–0.93 in the internal test cohort. In both cohorts, DSC and CCC were highest for LR-5. Segmentation and volumetry results are presented in detail in Table 3 and Fig. 5.

### Lesion detection

The sensitivity in detection was highest for lesions manually segmented as LR-5 in the training and internal test datasets (sensitivity_training_ = 0.85, sensitivity_test_ = 0.90) and lowest for LR-3 (sensitivity_training_ = 0.41, sensitivity_test_ = 0.40). PPV was highest among lesions segmented by nnU-Net as LR-5 (PPV_training_ = 0.94, PPV_test_ = 0.88) and lowest among LR-3 (PPV_training_ = 0.70) and LR-M (PPV_test_ = 0.67). Ground truth lesions below 10 mm were almost never predicted by nnU-Net. Lesions detection metrics are listed in Table 4.

**Table 4:** Lesion detection and LI-RADS classification metrics

	LR-3	LR-4	LR-5	LR-M
**Detection metrics ignoring predicted class** (lesions ≥ 10 mm)				
**Sensitivity**				
Tr	0.41 (0.37, 0.44)	0.68 (0.63, 0.72)	0.85 (0.81, 0.88)	0.75 (0.67, 0.82)
Ts	0.40 (0.34, 0.45)	0.65 (0.59, 0.70)	0.90 (0.85, 0.93)	0.80 (0.66, 0.90)
Ex	-	-	0.83	-
**PPV**				
Tr	0.70 (0.65, 0.75)	0.86 (0.81, 0.89)	0.94 (0.91, 0.96)	0.80 (0.72, 0.88)
Ts	0.79 (0.82, 0.85)	0.86 (0.80, 0.90)	0.88 (0.84, 0.92)	0.67 (0.52, 0.80)
Ex	-	-	0.90	-
**LI-RADS classification metrics of detected and manually marked lesions** (lesions ≥ 10 mm)				
**Sensitivity**				
Tr	0.57 (0.51, 0.63)	0.46 (0.40, 0.51)	0.75 (0.70, 0.79)	0.59 (0.49, 0.70)
Ts	0.66 (0.57, 0.75)	0.50 (0.43, 0.57)	0.80 (0.73, 0.85)	0.45 (0.3, 0.64)
Ex	-	-	0.80	-
**Specificity**				
Tr	0.86 (0.83, 0.89)	0.82 (0.78, 0.85)	0.78 (0.74, 0.81)	0.97 (0.96, 0.98)
Ts	0.88 (0.85, 0.91)	0.86 (0.82, 0.89)	0.78 (0.73, 0.82)	0.97 (0.95, 0.98)
Ex	-	-	0^a^	-
**PPV**				
Tr	0.59 (0.53, 0.66)	0.51 (0.44, 0.57)	0.64 (0.60, 0.70)	0.73 (0.62, 0.82)
Ts	0.59 (0.50, 0.67)	0.65 (0.58, 0.74)	0.69 (0.63, 0.74)	0.48 (0.32, 0.68)
Ex	-	-	0.89	-
**NPV**				
Tr	0.85 (0.82, 0.88)	0.79 (0.76, 0.82)	0.85 (0.82, 0.88)	0.95 (0.94, 0.97)
Ts	0.91 (0.88, 0.93)	0.76 (0.71, 0.80)	0.86 (0.82, 0.90)	0.96 (0.95, 0.98)
Ex	-	-	0^a^	-
**F1**				
Tr	0.58 (0.52, 0.63)	0.48 (0.43, 0.53)	0.69 (0.65, 0.73)	0.66 (0.57, 0.73)
Ts	0.62 (0.55, 0.70)	0.57 (0.50, 0.63)	0.74 (0.69, 0.78)	0.47 (0.33, 0.63)
Ex	-	-	0.84	-
**Kappa**				
Tr	0.44 (0.37, 0.50)	0.29 (0.22, 0.35)	0.50 (0.45, 0.56)	0.62 (0.53, 0.70)
Ts	0.51 (0.43, 0.61)	0.38 (0.29, 0.46)	0.56 (0.48, 0.62)	0.43 (0.29, 0.60)
Ex	-	-	-0.14	-

In parentheses: lower, upper bound of 95% confidence intervals. LR-3, LR-4, LR-5, *LR-M* LI-RADS categories, *mm* millimeters, *NPV* negative predictive value, *PPV* positive predictive value, *Tr* training cohort, *Ts* internal test cohort, *Ex* external test cohort. Bold: metric or metric category. ^a^No true negative samples

### LI-RADS classification of detected lesions

When comparing the LI-RADS category of the manually segmented ground truth lesions ≥ 10 mm and corresponding predicted lesions from the nnU-Net, sensitivity, and F1 values were highest for LR-5 lesions (sensitivity_training_ = 0.75, sensitivity_test_ = 0.80, F1_training_ = 0.69, F1_test_ = 0.74), while for other LI-RADS categories, the values ranged between 0.50–0.66 in the two cohorts. Specificity and NPV were high for all LI-RADS categories (Specificity ≥ 0.78, NPV ≥ 0.76) and highest for LR-M lesions (specificity_training_ = 0.97, specificity_test_ = 0.97, NPV_training_ = 0.95, NPV_test_ = 0.96). Kappa values were highest for LR-M and LR-5 lesions (κ_training_ = 0.62, κ_test_ = 0.56). Larger LR-5 lesions were more often categorized accurately and mislabeled predicted lesions were most frequently misclassified as the neighboring LI-RADS category (see Fig. 3). Classification metrics are listed in Table 4. Confusion matrices of the training and internal test datasets with example lesions from the internal test dataset with corresponding segmentations are shown in Fig. 3.

### Occlusion sensitivity analysis

**Fig. 6:**
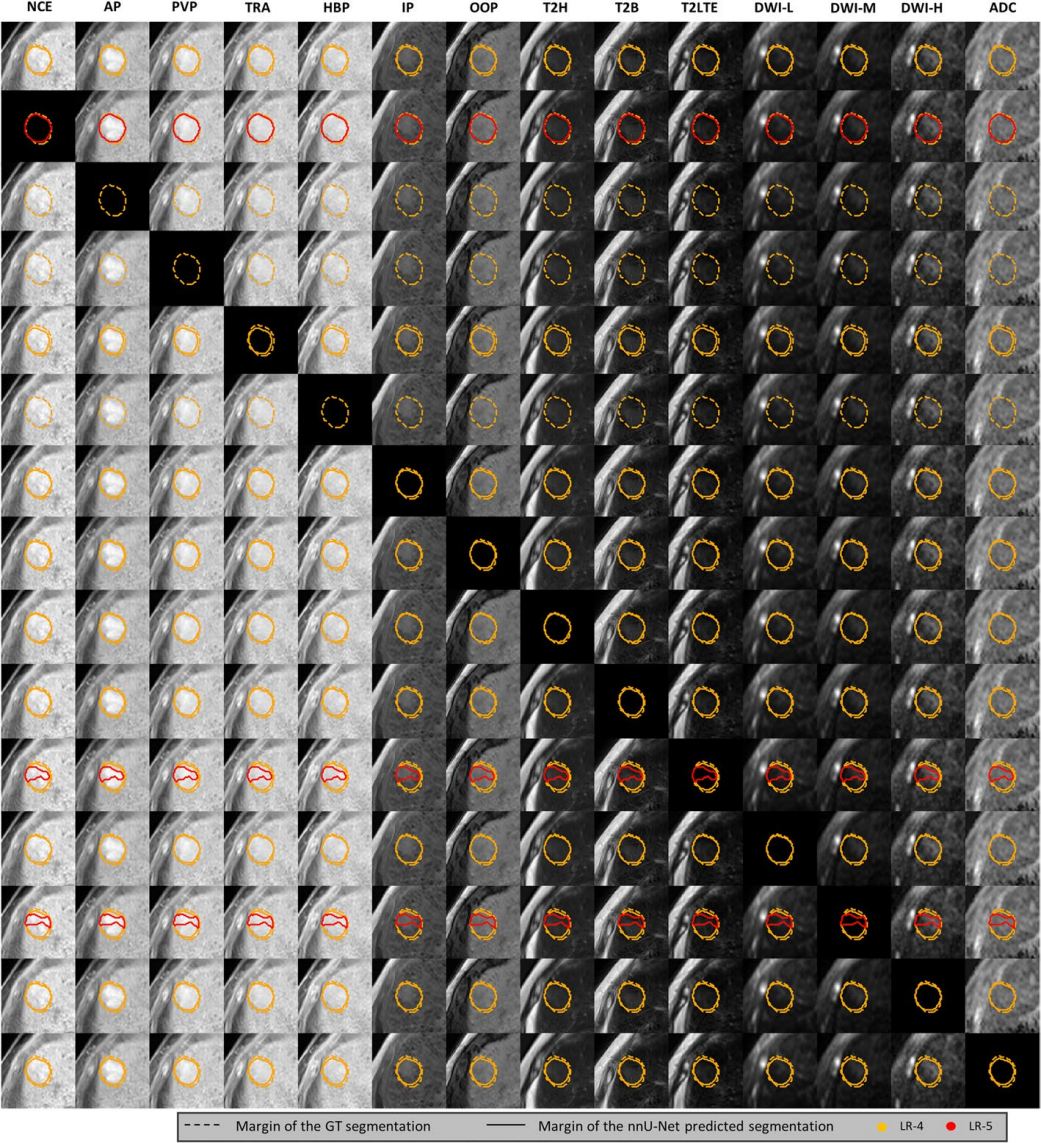
Example case illustration of the effects of input image removal on the output segmentations. Black images indicate input image replacement with an image containing only zero values. Input channels in order: NCE—non-contrast T1; AP, PVP, TRA—arterial, portal venous, transitional phase contrast-enhanced T1; HBP – hepatobiliary phase contrast-enhanced T1; IP, OOP – in- and out-of-phase T1-weighted sequences; T2H – T2-weighted HASTE; T2B – T2-weighted BLADE; T2LTE – Multiple types of T2-weighted images with longer time to echo; DWI-L, DWI-M, DWI-H – diffusion-weighted imaging with three increasing b-value ranges; ADC – apparent diffusion coefficient maps. GT: ground truth; LR-3, LR-4, LR-5: LI-RADS categories

In the occlusion sensitivity analysis, we evaluated the contribution of each input channel by replacing each channel, one at a time, with an image of all zeros (Fig. 6). We then calculated the percent change within each metric for that given channel. The three most important inputs contributing to lesion detection (% change of sensitivity) were HBP (-85.3%), AP, NCE for LR-3; AP (-77.4%), HBP, NCE for LR-4; AP (-42.1%), HBP, PVP for LR-5 and AP (-48.5%), HBP, PVP for LR-M.

Lesion segmentation quality (instance segmentation DSC) was reduced by omission of HBP (-37.5), AP and NCE for LR-3; AP (-35.3%), PVP and HBP for LR-4; AP (-16,8%), HBP (-16.4%) and NCE for LR-5; and NCE, AP, T2H, HBP, PVP (ranging between -7.6% and -4.7%) for LR-M (the removal of the majority of the image groups increased DSC for LR-M lesions).

**Fig. 7:**
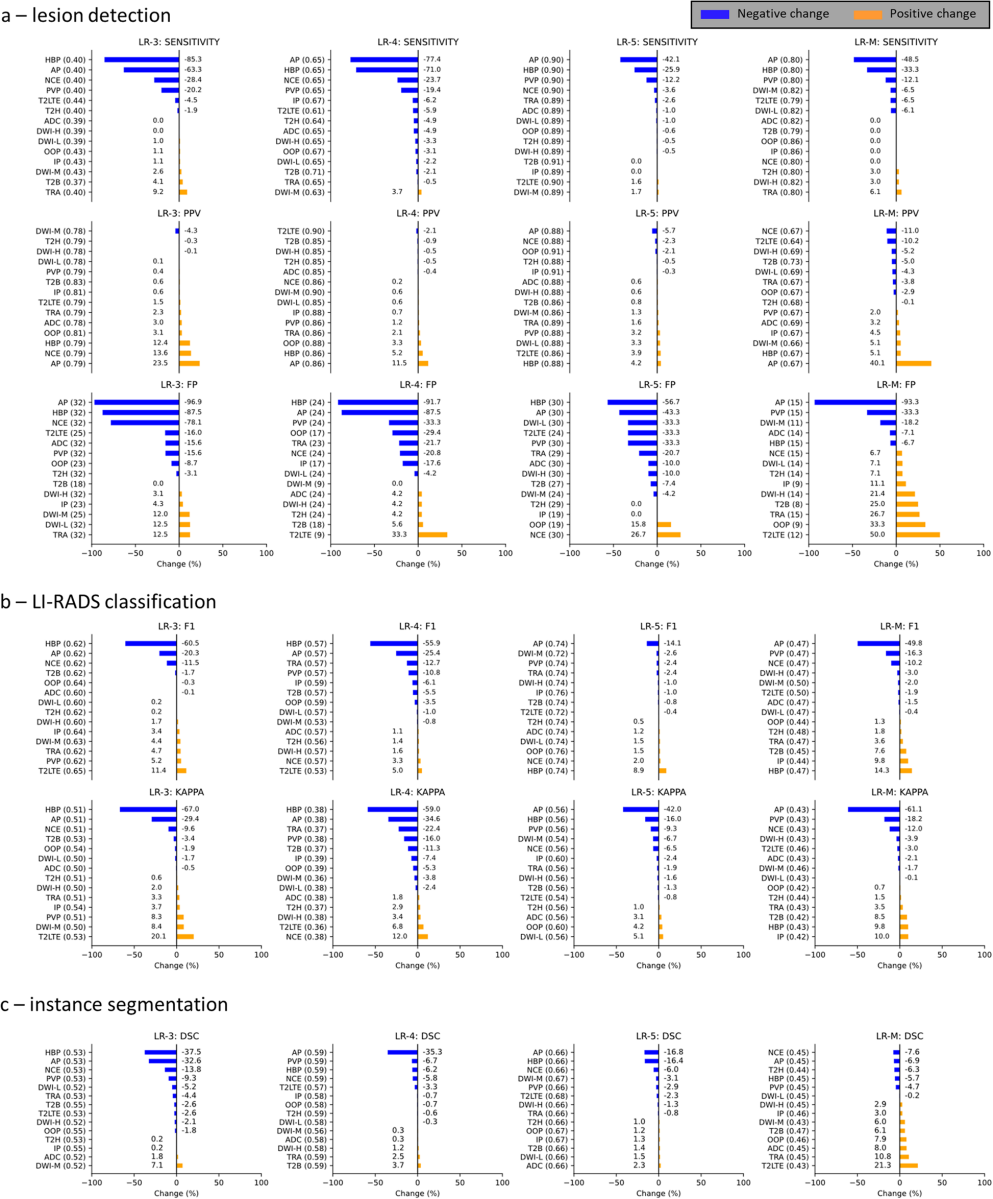
Ranked changes after replacing each input image group with an image containing only zero values. Horizontal axes: percentage of change in the given metric (titles) compared to the same set of cases where the given input image type was present. Vertical axes: U-Net input channel ordered from most negative to most positive change per metric from top to bottom. Original values of each metric are noted in parentheses after the abbreviation of the channel. LR-3, LR-4, LR-5, LR-M: included LI-RADS categories; DSC: Sørensen–Dice coefficient; FP: false positives; PPV: positive predictive value. Input channels in order: NCE—pre-contrast T1; AP, PVP, TRA—arterial, portal venous, transitional phase contrast-enhanced T1; HBP – hepatobiliary phase contrast-enhanced T1; IP, OOP – in- and out-of-phase T1-weighted sequences; T2H – T2-weighted HASTE; T2B – T2-weighted BLADE; T2LTE – multiple types of T2-weighted images with longer time to echo; DWI-L, DWI-M, DWI-H – diffusion-weighted imaging with three increasing b-value ranges; ADC – apparent diffusion coefficient maps

Based on the percentage change of F1 scores, HBP (-60.5%, -55.9%) and AP (-20.3%, -25.4%) had the highest impact on LR-3 and LR-4 lesion classification, followed by NCE for LR-3 and TRA for LR-4. The most influential group for LR-5 classification was AP (-14.1%), other groups showed minor contributions or increased the F1 score, which is possibly due to the reduction in the detection of LR-3 and LR-4 lesions. For LR-M, the most impactful groups were AP (-49.8%), PVP and NCE. The ranked changes for each metric are shown in Fig. 7. An example case is shown in Fig. 6.

### External validation

One examination from the external test dataset was excluded due to inadequate image quality. Almost all lesions in the external cohort were categorized as LR-5. Sensitivity and PPV in lesion detection were 0.83 and 0.90, respectively. The F1 score for LI-RADS classification of predicted LR-5 lesions was 0.84. Per lesion, the median DSC was 0.84 (0.65, 0.87). Detailed results are shown in Tables 3 and 4 and Fig. 4.

## Discussion

In the present study, an automatic DCE-MRI segmentation model for hepatocellular carcinoma (HCC) risk assessment was developed using nnU-Net. The model showed moderate agreement in the classification of LR-5 lesions compared to a gold standard expert read and excellent agreement in LR-5 volume prediction. Whole liver segmentation allowed for the exclusion of erroneously segmented lesions outside the liver boundaries. For this, the initial segmentations of a pre-trained liver segmentation model could be improved by further training with nnU-Net by including more images per examination. Co-registration of images made segmentations transferable to all included MRI sequences. By occluding the images, the contribution of each image group to the final lesion segmentation and classification was measured. The results from our segmentation model were validated using an external dataset composed of MRIs with extracellular and hepatocyte-specific contrast agents.

DL-based algorithms such as the one from the present study could potentially alleviate some of the limitations of LI-RADS [11]. Although LI-RADS reduced HCC reporting variability compared to non-standardized reporting, it did not eliminate it [7]. Interreader inconsistency is common, can have a strong impact on patient management, and partly be attributed to the complexity of LI-RADS [26]. Standardized LI-RADS assessment can be more time-consuming than narrative reporting [10]. The comparatively good performance of our segmentation model in the detection and segmentation of LR-5 lesions shows that DL-based algorithms could assist in lesion classification, especially for inexperienced radiologists in cases with widespread disease or high-volume reporting [10]. The kappa value of LR-5 lesions from our model versus expert opinion (0.56) was almost equal to the reported kappa of twenty untrained radiologists versus expert opinion (0.57), but lower than the kappa of the same twenty radiologists after a special LI-RADS training (0.77) [27]. In the present study, LR-3, LR-4 and LR-M lesions were more often discordant in detection and classification which is in line with discordances in the assignment of these categories by radiologists in previous studies [26, 27]. Notably, the performance of untrained radiologists for the assignment of LR-4 and LR-M lesions was within the same range as our model’s performance but improved after a special LI-RADS training [27]. The greater variability of the LR-3 and LR-4 categories can be explained by the larger amount of possible imaging feature combinations that can lead to LR-3/LR-4 assignments, especially when also considering ancillary features [28]. In the case of ambiguity in LI-RADS features, tie-breaking rules often lead to the categorization of equivocal lesions as LR-3/LR-4 [28]. Moreover, processes in the background liver parenchyma such as perfusion alterations that are often detected by MRI can be mistakenly diagnosed as LR-3, instead of LR-2 [28]. Disagreement regarding LR-M lesions is partly explainable by the various differential-diagnostic possibilities such as intrahepatic cholangiocarcinoma, hepatocholangiocarcinoma, atypical HCC, metastasis, lymphoma, and multiple benign entities [29].

The satisfactory performance of our model in LI-RADS category assignment coupled with high sensitivity and PPV for lesion detection suggests several potential use cases. It could be used for automated secondary analysis of MRI cases where lesion assessment in the original report was not according to (the newest version of) LI-RADS. The automation of the segmentation enables large-scale analyses for local or multicenter research projects and clinical investigations. Precise measurements of tumor volume facilitate intra- and interindividual comparisons of tumor burden for response assessment. Also, the extraction of radiomics features of liver lesions for the prediction of histopathological features and prognostication is made possible by our segmentation model [30].

Multiple research groups have published machine learning (ML)- and DL-based studies for automated liver lesion segmentation and/or classification in patients at risk for HCC. Several semi-automatic and automatic segmentation and (LI-RADS) classification approaches for MRI have been reported. However, these approaches are limited by either the need for human annotation for segmentation [31], for classification [32, 33], or they were only tested on a small number of unequally distributed lesions per LI-RADS category, with a disproportionate prevalence of LR-5 lesions [34]. Our approach for automated LI-RADS segmentation/detection/classification differs from the above-mentioned studies. Our model is a fully automated end-to-end semantic segmentation model without a separate assessment of individual imaging features in an interim step. This approach, to our knowledge, is unique in the RADS literature. Our approach allows for a separate evaluation of the effect of individual imaging features on LI-RADS category assignment, although not by analysis of the features themselves but by modification of the images that may contain them. Our segmentation model was trained and tested on a large well-characterized radiological dataset, consisting of heterogeneously acquired MRI scans, comprising lesions with differences in size and texture within the same LI-RADS category. We also show that the nnU-Net pipeline scales well to MRI-based tasks that are more complex than most previously reported use cases. As a byproduct of our analyses, we have shown that nnU-Net improves liver segmentation quality when less accurate liver segmentations are provided as ground truth along with additional input images in multiple input channels.

Limitations of our study include the determination of the gold-standard segmentation and LI-RADS classification by only one expert radiologist, the lack of separate evaluation of distinct LI-RADS features, the incomplete implementation of LI-RADS categories (only LR-3–5 and LR-M were marked), the lack of correlation with histopathological diagnosis, the lack of correlation of the (automated) classification results with the etiology of the underlying liver disease (e.g. alcohol, chronic virus hepatitis), and the use of a single type of hepatocyte-specific contrast agent in the internal datasets. Future studies addressing these limitations would be beneficial.

## Conclusion

In conclusion, we proposed and evaluated a simplified approach for the DL-based automation of LI-RADS v2018. We showed that self-configuring semantic segmentation pipelines, like nnU-Net, can be used to detect LR-5 lesions with high sensitivity and PPV and directly extract LI-RADS classification results which show moderate agreement, PPV, and specificity compared to expert classification. Such models have a wide range of downstream use cases from research, such as data exploration as demonstrated on an external cohort, to clinical decision support and quality assurance systems.

## Supplementary Information

Additional file 1. Supplementary Methods.

Additional file 2. Dataset fingerprint file used by nnU-Net.

Additional file 3. Inference information for nnU-Net.

Additional file 4. Inference instructions.

Additional file 5. Plans file generated by nnU-Net.

Additional file 6. Learning curves generated by nnU-Net (2d, 3d_fullres).

Additional file 7. Learning curves generated by nnU-Net (3d_lowres, 3d_cascade_fullres).

## Data Availability

The datasets used and/or analysed during the current study are available from the corresponding author on reasonable request.

## Abbreviations

ADC: Apparent diffusion coefficient maps
ADIT: Automated DICOM Transfer
AP: Arterial phase contrast-enhanced T1-weighted
BG: Background
CCC: Concordance correlation coefficient
CHILD-A, -B, -C: Child-Turcott-Pugh scores
DCE-MRI: Dynamic contrast-enhanced magnetic resonance imaging
DL: Deep learning
DS: Dataset
DSC: Sørensen–Dice coefficient
DWI: Diffusion-weighted imaging
EOB-MRI: Gadoxetate disodium-enhanced magnetic resonance imaging
Ex: External test dataset
FN: False negative
FP: False positive
GT: Ground truth
HBP: Hepatobiliary phase contrast-enhanced T1-weighted
HBV: Hepatitis B virus
HCC: Hepatocellular carcinoma
HCV: Hepatitis C virus
IP: In-phase T1-weighted
LI-RADS: Liver Imaging Reporting And Data System
ML: Machine Learning
MV: Volume calculated from manual segmentations
NASH: Nonalcoholic steatohepatitis
NCE: Non-contrast T1-weighted
NIfTI: Neuroimaging Informatics Technology Initiative
NPV: Negative predictive value
OOP: Out-of-phase T1-weighted
PPV: Positive predictive value
PV: Volume calculated from U-Net predicted segmentations
PVP: Portal venous phase contrast-enhanced T1-weighted
RV: Reference volume
T2B: T2-weighted periodically rotated overlapping parallel lines with enhanced reconstruction (PROPELLER—BLADE®, Siemens Healthcare)
T2H: T2-weighted Half-Fourier Acquisition Single-shot Turbo spin Echo (HASTE)
T2LTE: T2-weighted image with longer time to echo
TN: True negative
TP: True positive
Tr: Training dataset
TRA: Transitional phase contrast-enhanced T1-weighted
Ts: Internal test dataset
