# Supplementary material for: LI-RADS-based hepatocellular carcinoma risk mapping using contrast-enhanced MRI and self-configuring deep learning
Source: Cancer Imaging. 2025 Mar 17;25:36. doi: 10.1186/s40644-025-00844-6 (PMC11912691; doi:10.1186/s40644-025-00844-6)
Supplement: Supplementary file 1 — Additional file 1. Supplementary Methods. [file 40644_2025_844_MOESM1_ESM.docx]

**Supplementary Methods**

Image co-registration:

The pre-contrast (native) T1-weighted MRI images are used as fixed images. T1-weighted axial images are co-registered using BSpline, coronal images, and all T2-weighted, DWI and ADC images are co-registered using rigid registration.

Deep learning model development:

Model training was performed with the nnU-Net pipeline with fivefold cross-validation run with the 3d_lowres, 3d_fullres and 3d_fullres_cascaded trainers for 1000 epochs per fold, the 2d U-Net was only trained for one fold due to low performance metrics. The trained models are ensembled per trainer class and their cross-validated results are compared to select a final ensemble of models. As a result, 3d_lowres, and 3d_fullres_cascaded networks were ensembled (5-5 models, all with 14 input channels) and, combined with post-processing, was determined as the best performing model combination, which was then used for inference. Network weights were randomly initialized, and no pre-trained weights were used for training.

Important software libraries, frameworks, and packages not listed in the article (version):

Training, inference:

Python (3.10) – packages:

- nnunetv2 (2.2)

Dataset creation, statistical evaluation, figures:

Python (3.9.12) – packages:

- dicom2nifti (2.4.3)
- matplotlib (3.5.1)
- numpy (1.26.0)
- pandas (2.2.1)
- pydicom (2.3.0)
- scikit-learn (1.0.2)
- scipy (1.12.0)
- SimpleITK (2.3.1)
