# Supplementary figures and images for: LI-RADS-based hepatocellular carcinoma risk mapping using contrast-enhanced MRI and self-configuring deep learning
Source: Cancer Imaging. 2025 Mar 17;25:36. doi: 10.1186/s40644-025-00844-6 (PMC11912691; doi:10.1186/s40644-025-00844-6)

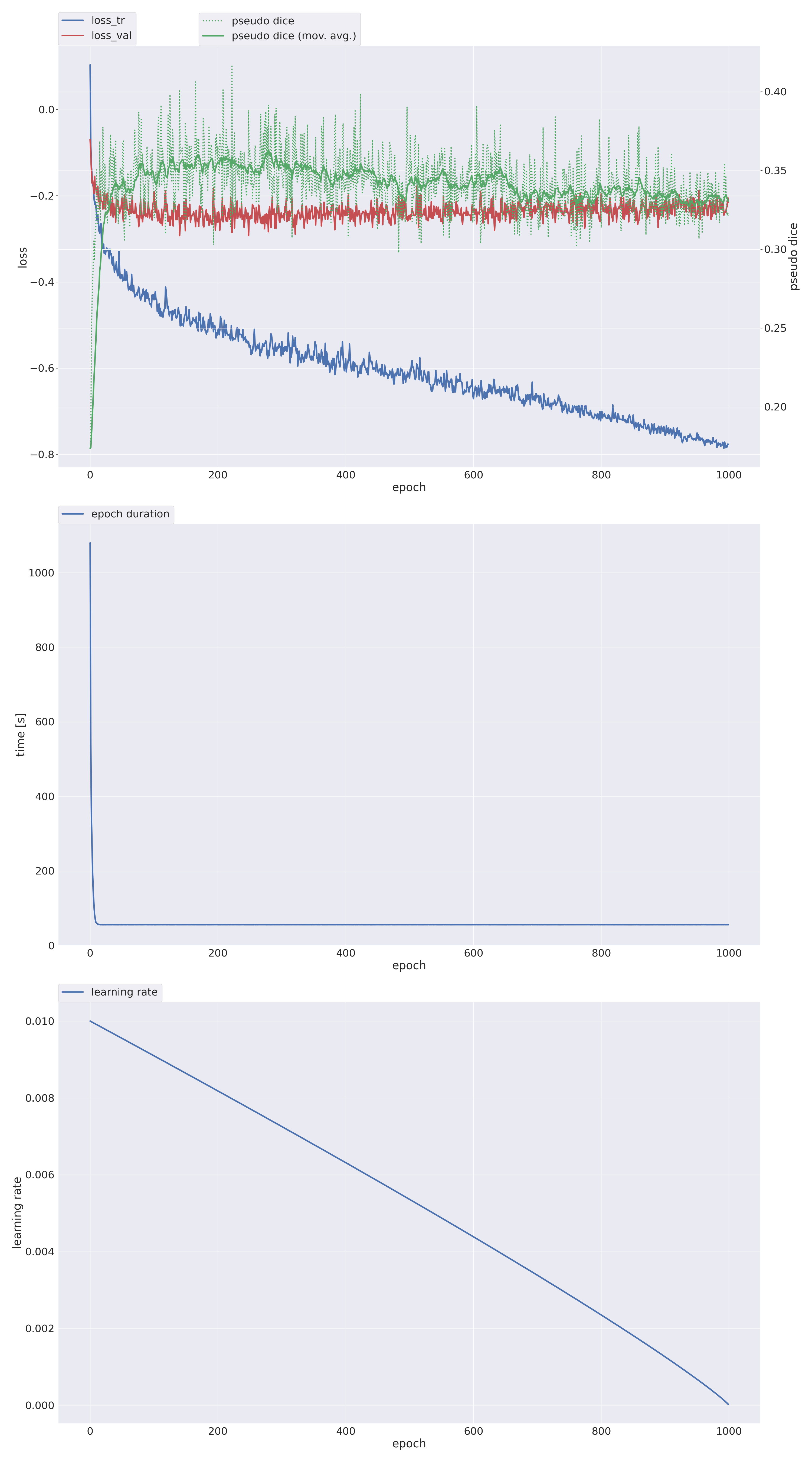

Supplement: Supplementary file 6 — Additional file 6. Learning curves generated by nnU-Net (2d, 3d_fullres). [file 40644_2025_844_MOESM6_ESM.zip › learning_curves_1/2d-fold_0.png]

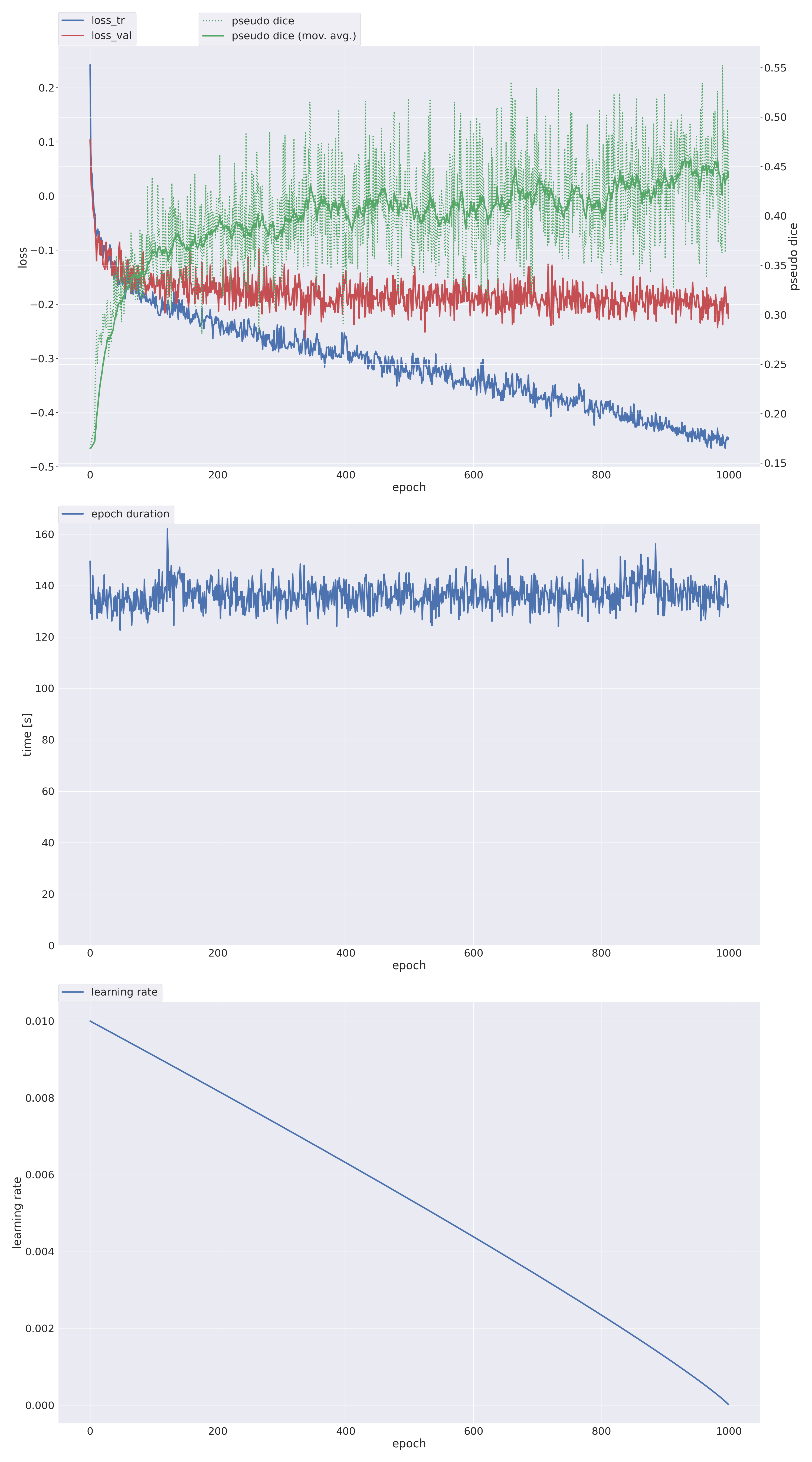

Supplement: Supplementary file 6 — Additional file 6. Learning curves generated by nnU-Net (2d, 3d_fullres). [file 40644_2025_844_MOESM6_ESM.zip › learning_curves_1/3d_fullres-fold_0.png]

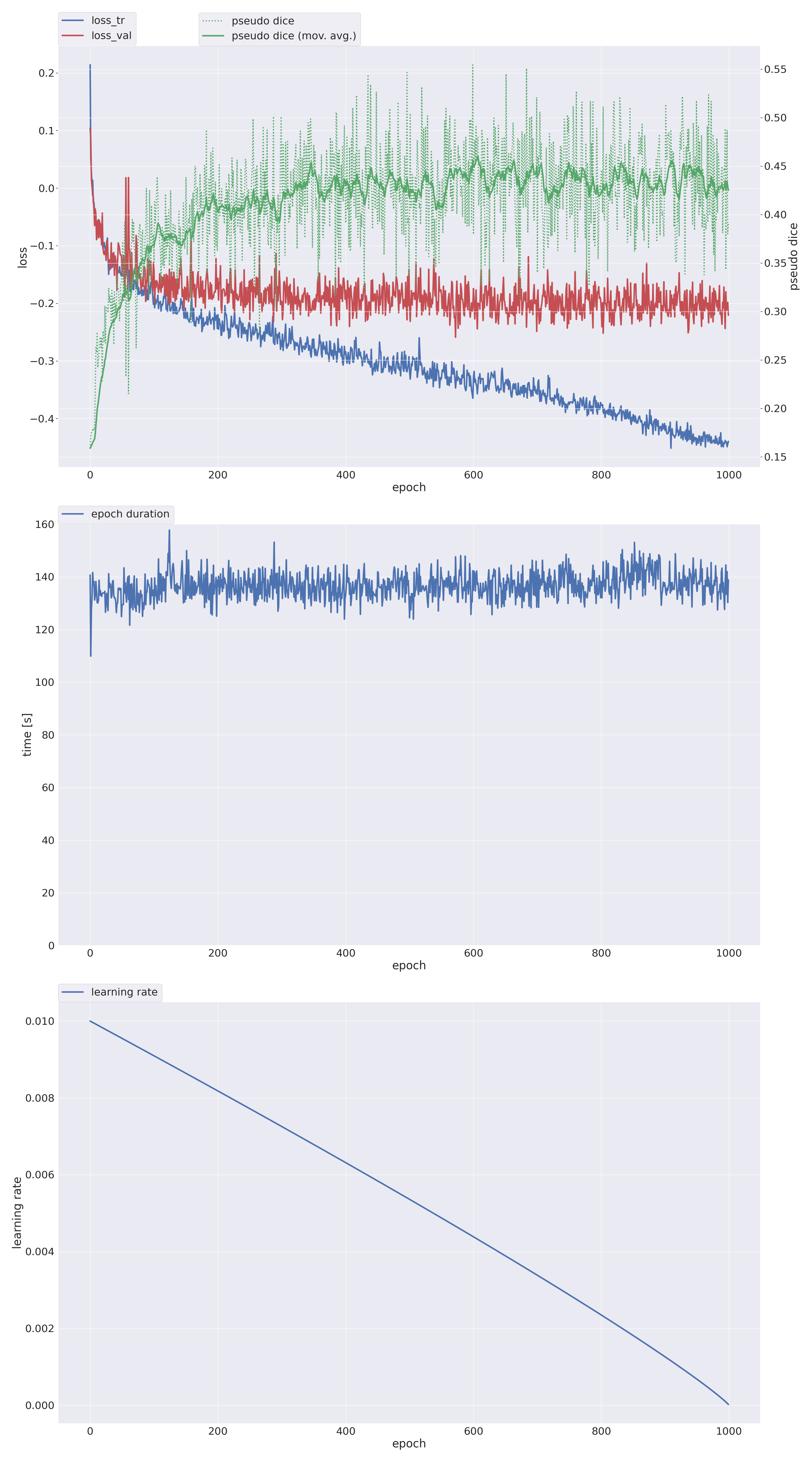

Supplement: Supplementary file 6 — Additional file 6. Learning curves generated by nnU-Net (2d, 3d_fullres). [file 40644_2025_844_MOESM6_ESM.zip › learning_curves_1/3d_fullres-fold_1.png]

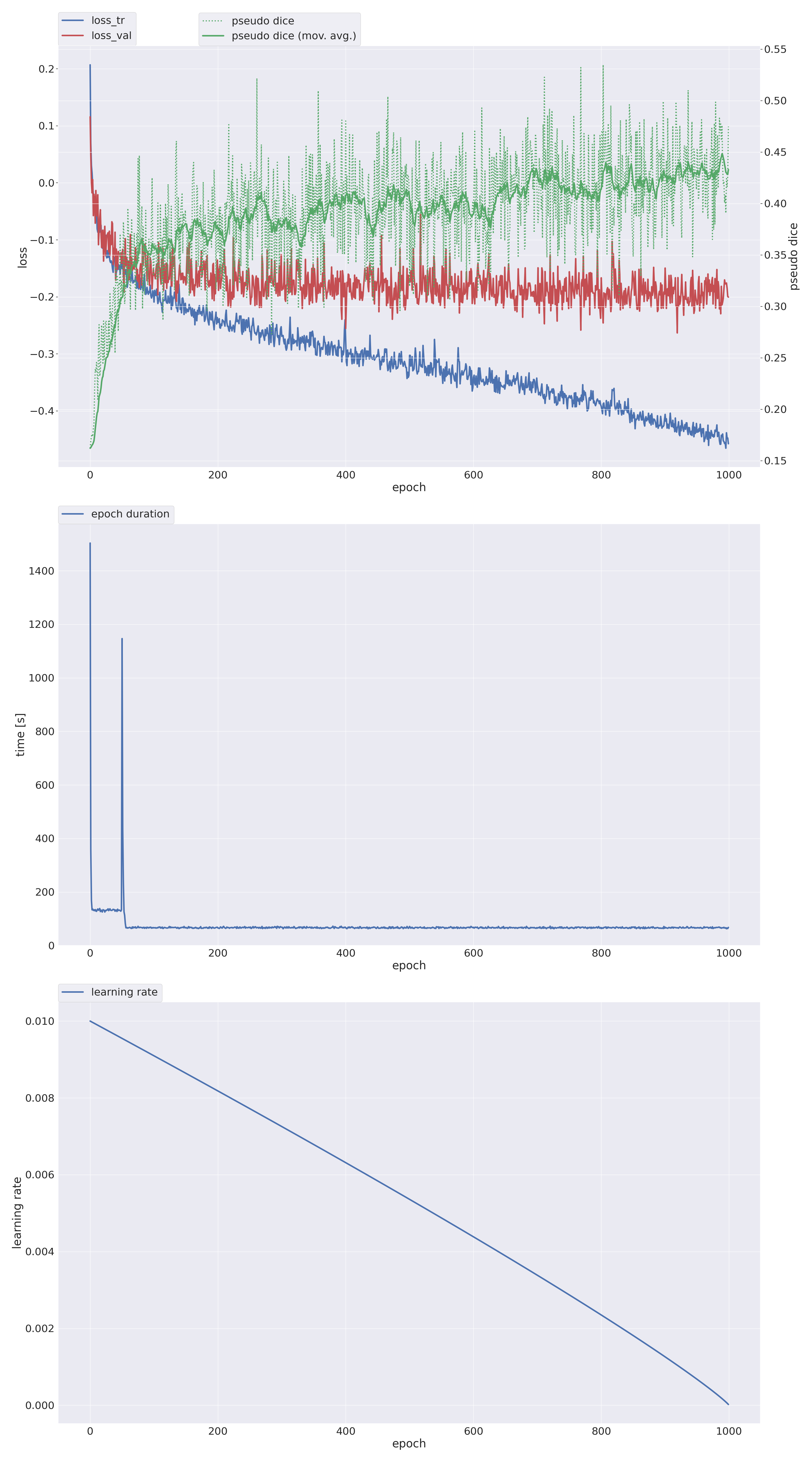

Supplement: Supplementary file 6 — Additional file 6. Learning curves generated by nnU-Net (2d, 3d_fullres). [file 40644_2025_844_MOESM6_ESM.zip › learning_curves_1/3d_fullres-fold_2.png]

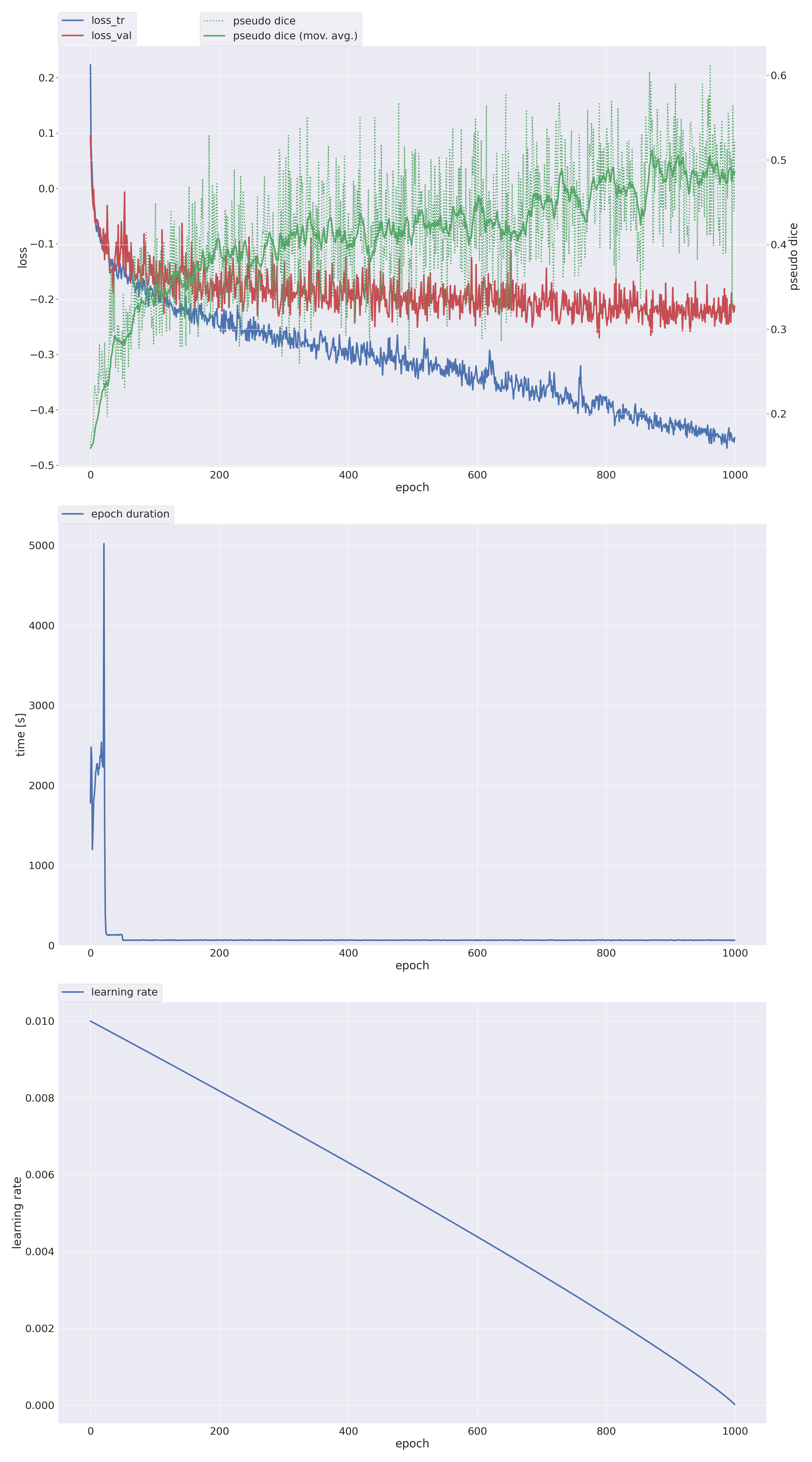

Supplement: Supplementary file 6 — Additional file 6. Learning curves generated by nnU-Net (2d, 3d_fullres). [file 40644_2025_844_MOESM6_ESM.zip › learning_curves_1/3d_fullres-fold_3.png]

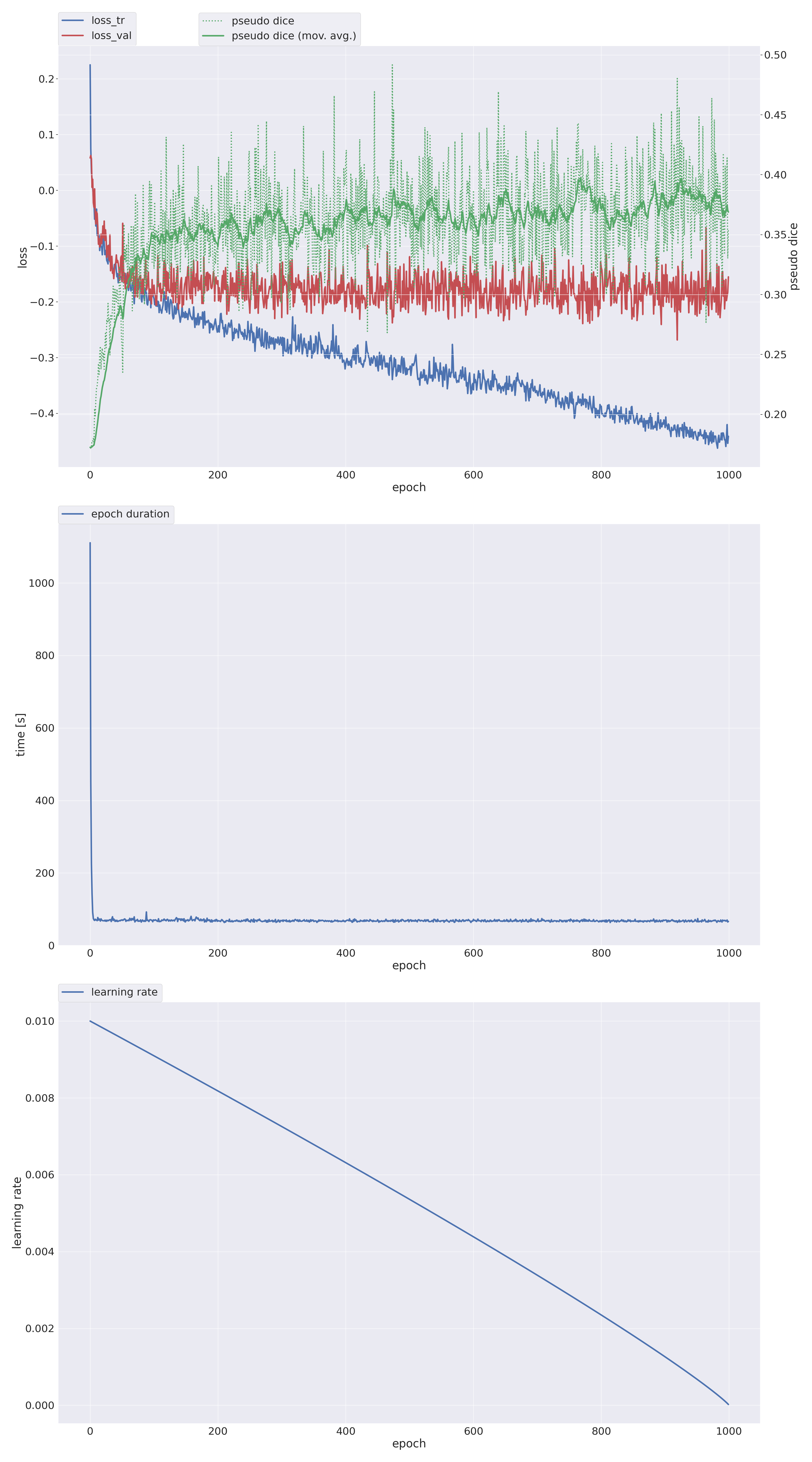

Supplement: Supplementary file 6 — Additional file 6. Learning curves generated by nnU-Net (2d, 3d_fullres). [file 40644_2025_844_MOESM6_ESM.zip › learning_curves_1/3d_fullres-fold_4.png]

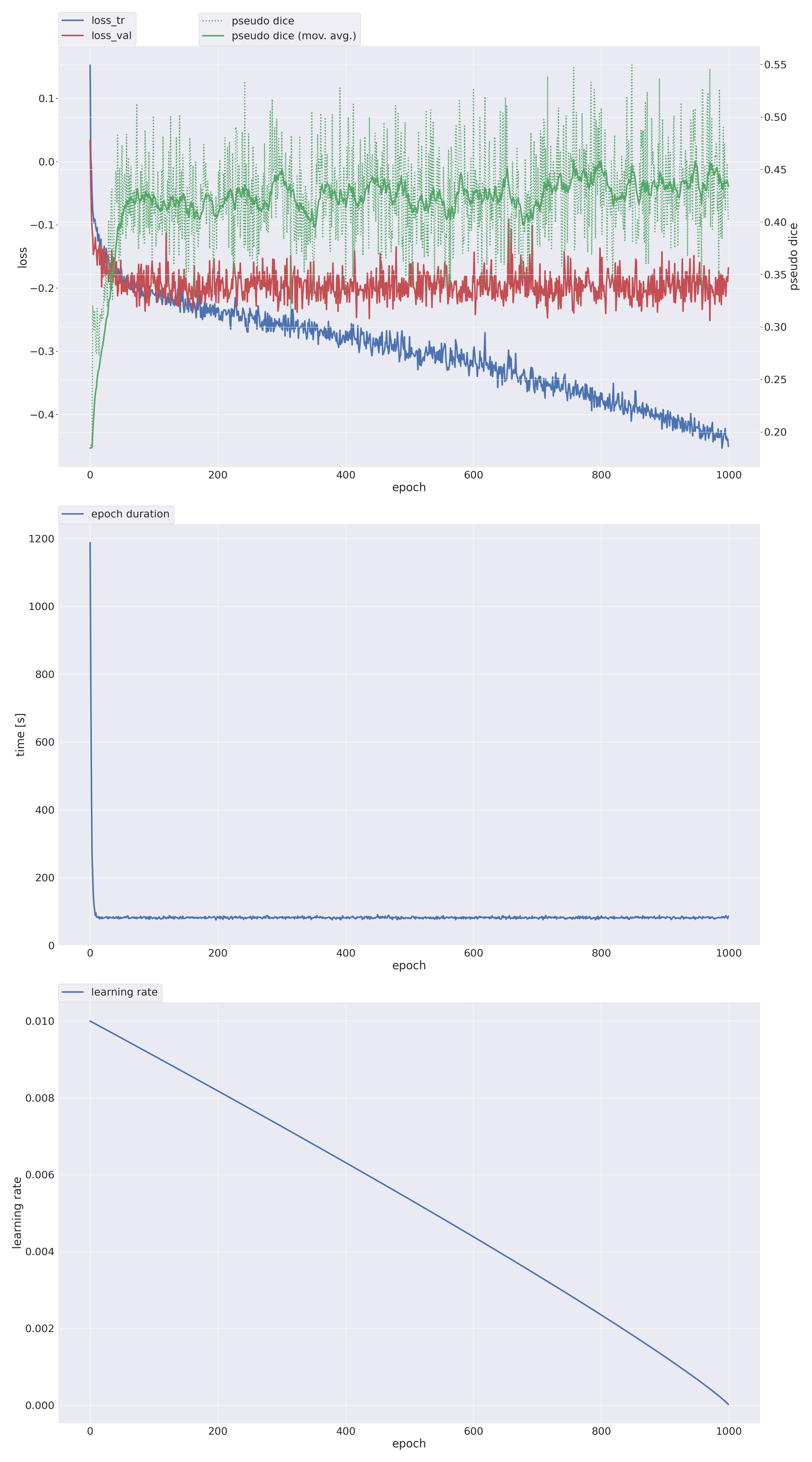

Supplement: Supplementary file 7 — Additional file 7. Learning curves generated by nnU-Net (3d_lowres, 3d_cascade_fullres). [file 40644_2025_844_MOESM7_ESM.zip › learning_curves_2/3d_cascade_fullres-fold_0.png]

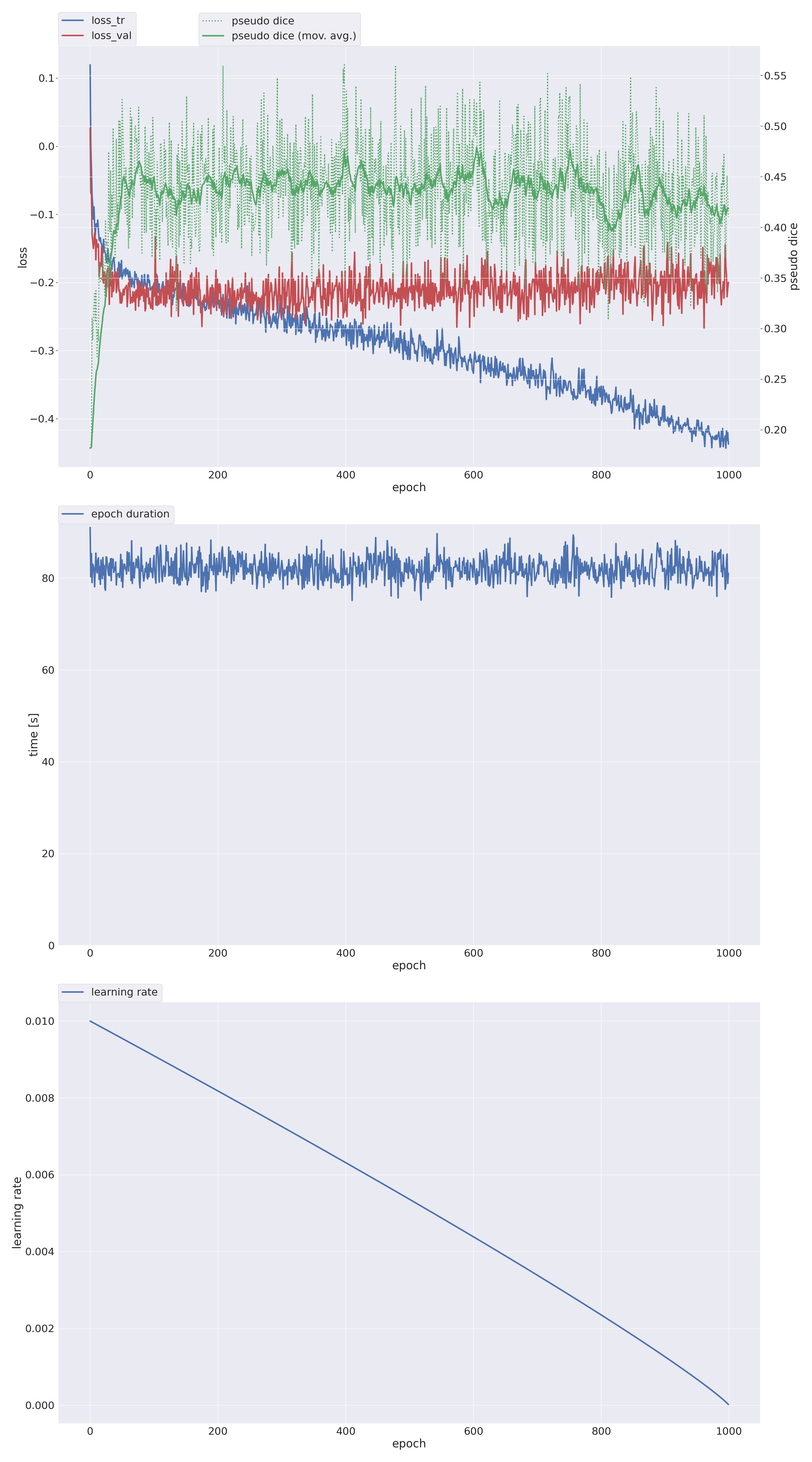

Supplement: Supplementary file 7 — Additional file 7. Learning curves generated by nnU-Net (3d_lowres, 3d_cascade_fullres). [file 40644_2025_844_MOESM7_ESM.zip › learning_curves_2/3d_cascade_fullres-fold_1.png]

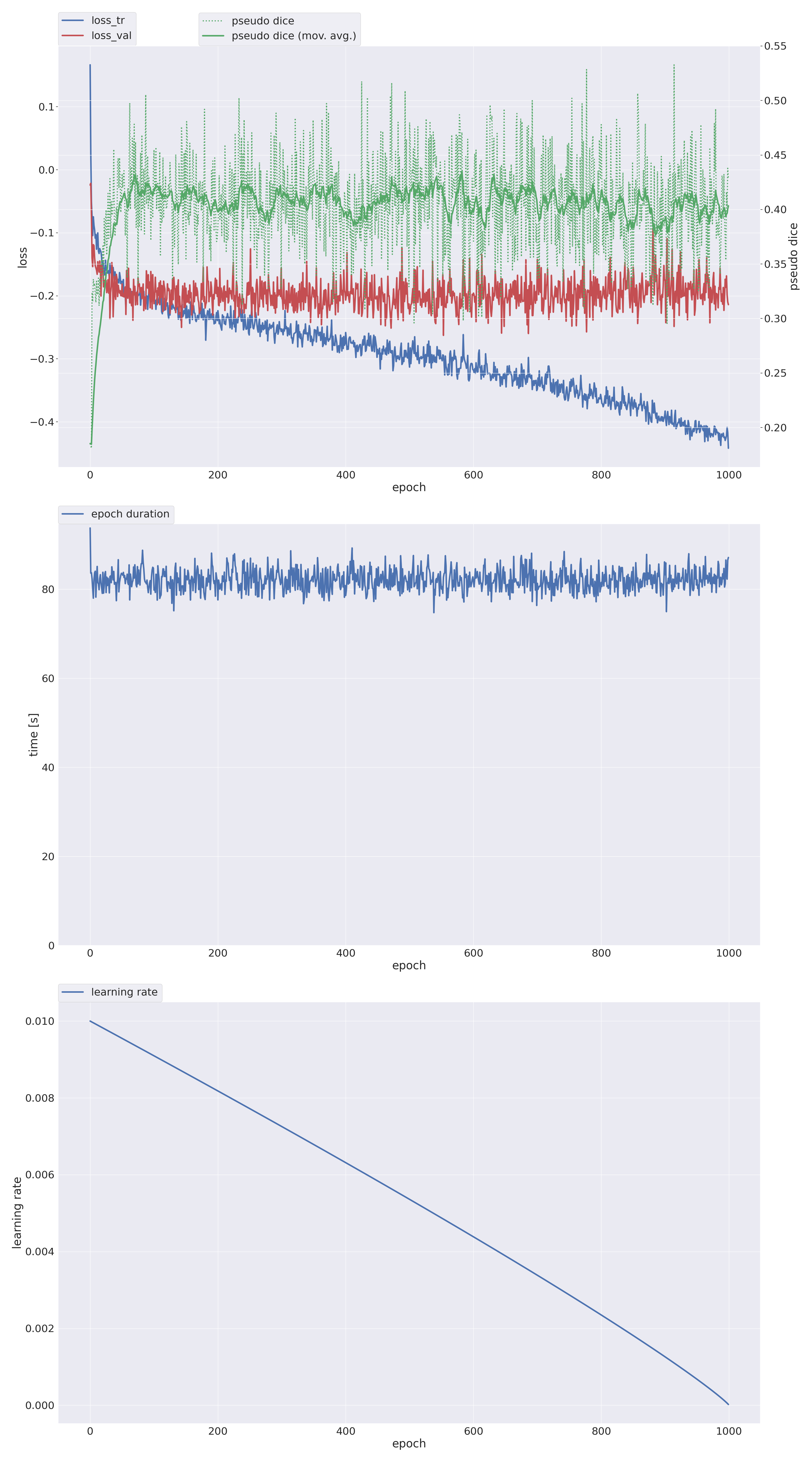

Supplement: Supplementary file 7 — Additional file 7. Learning curves generated by nnU-Net (3d_lowres, 3d_cascade_fullres). [file 40644_2025_844_MOESM7_ESM.zip › learning_curves_2/3d_cascade_fullres-fold_2.png]

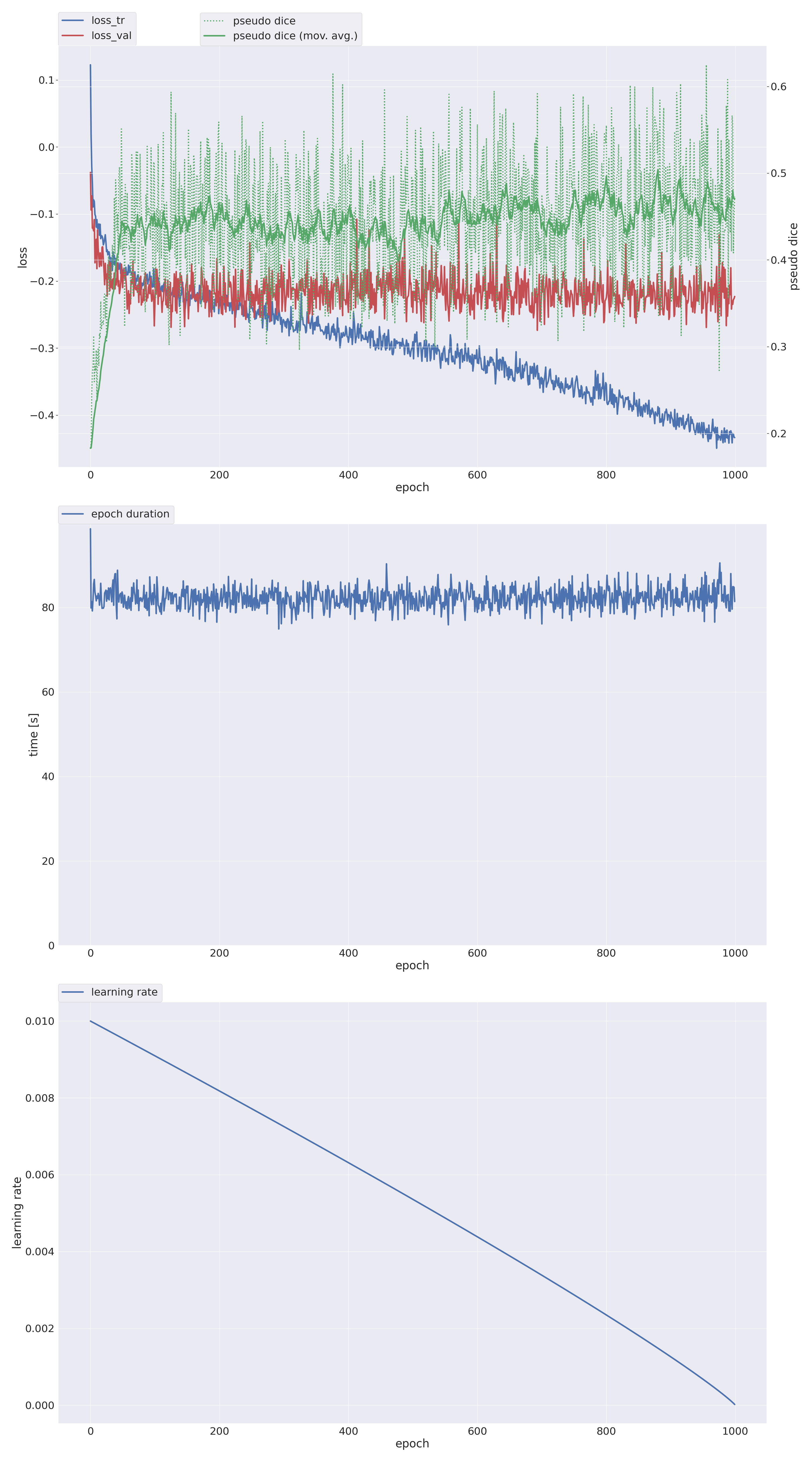

Supplement: Supplementary file 7 — Additional file 7. Learning curves generated by nnU-Net (3d_lowres, 3d_cascade_fullres). [file 40644_2025_844_MOESM7_ESM.zip › learning_curves_2/3d_cascade_fullres-fold_3.png]

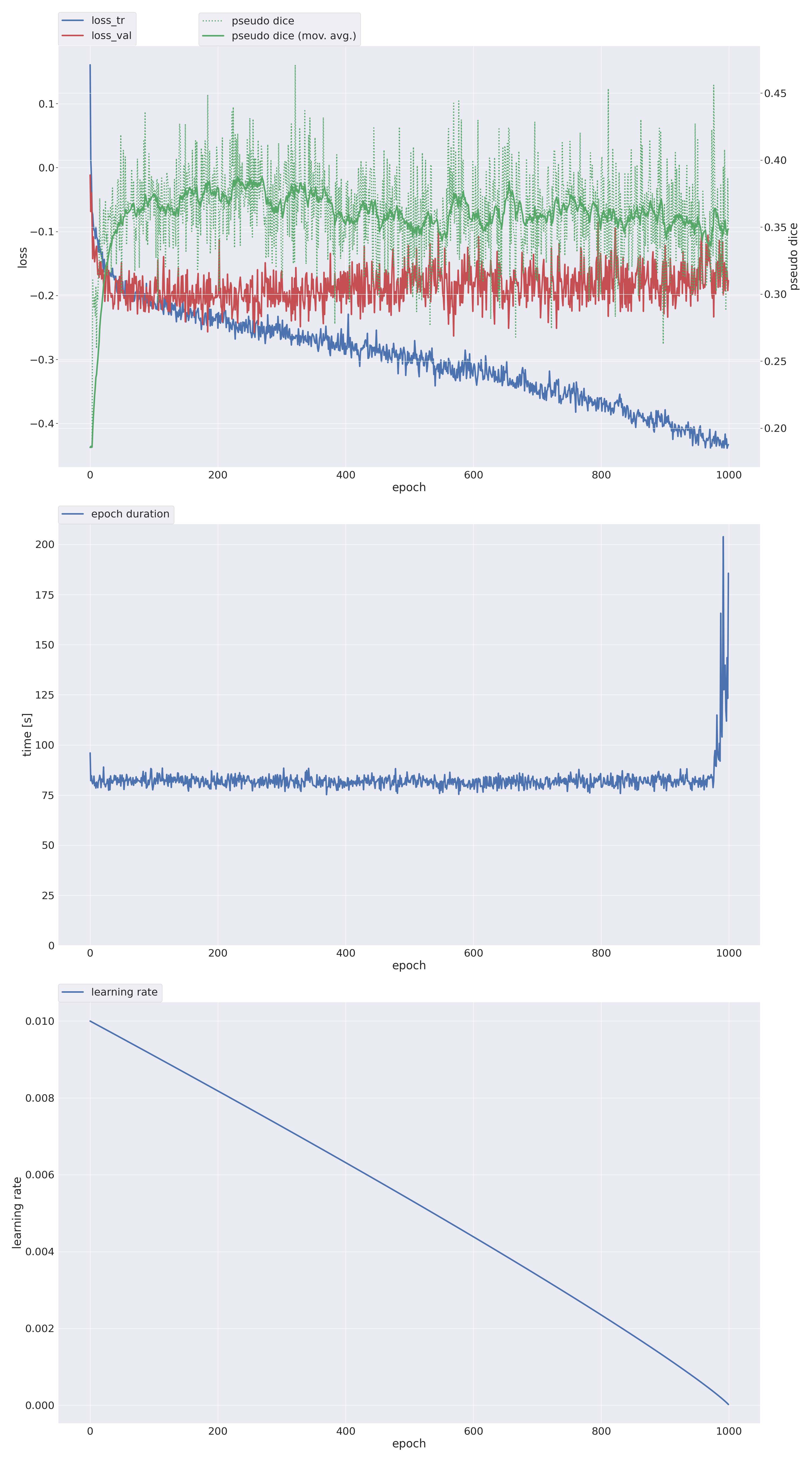

Supplement: Supplementary file 7 — Additional file 7. Learning curves generated by nnU-Net (3d_lowres, 3d_cascade_fullres). [file 40644_2025_844_MOESM7_ESM.zip › learning_curves_2/3d_cascade_fullres-fold_4.png]

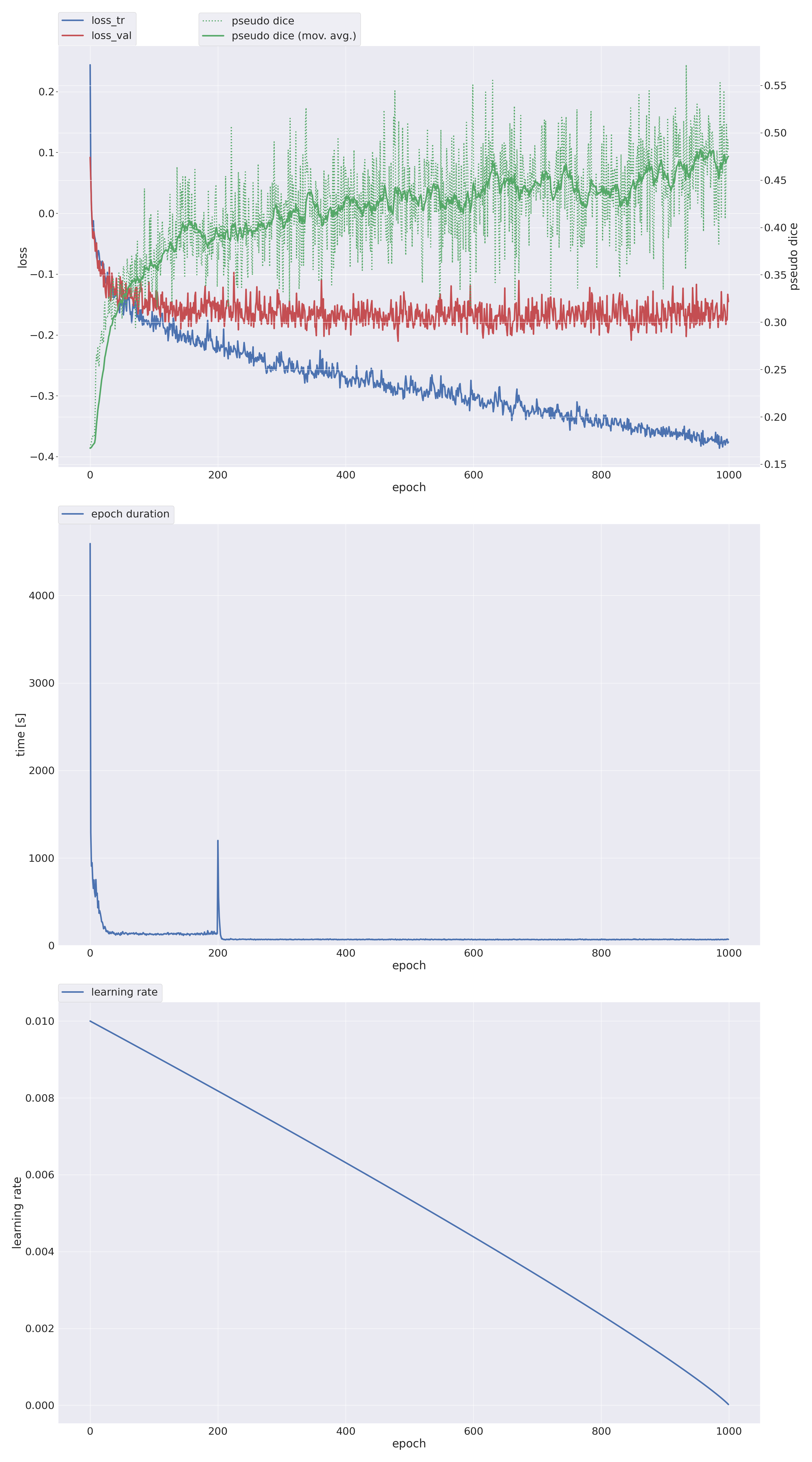

Supplement: Supplementary file 7 — Additional file 7. Learning curves generated by nnU-Net (3d_lowres, 3d_cascade_fullres). [file 40644_2025_844_MOESM7_ESM.zip › learning_curves_2/3d_lowres-fold_0.png]

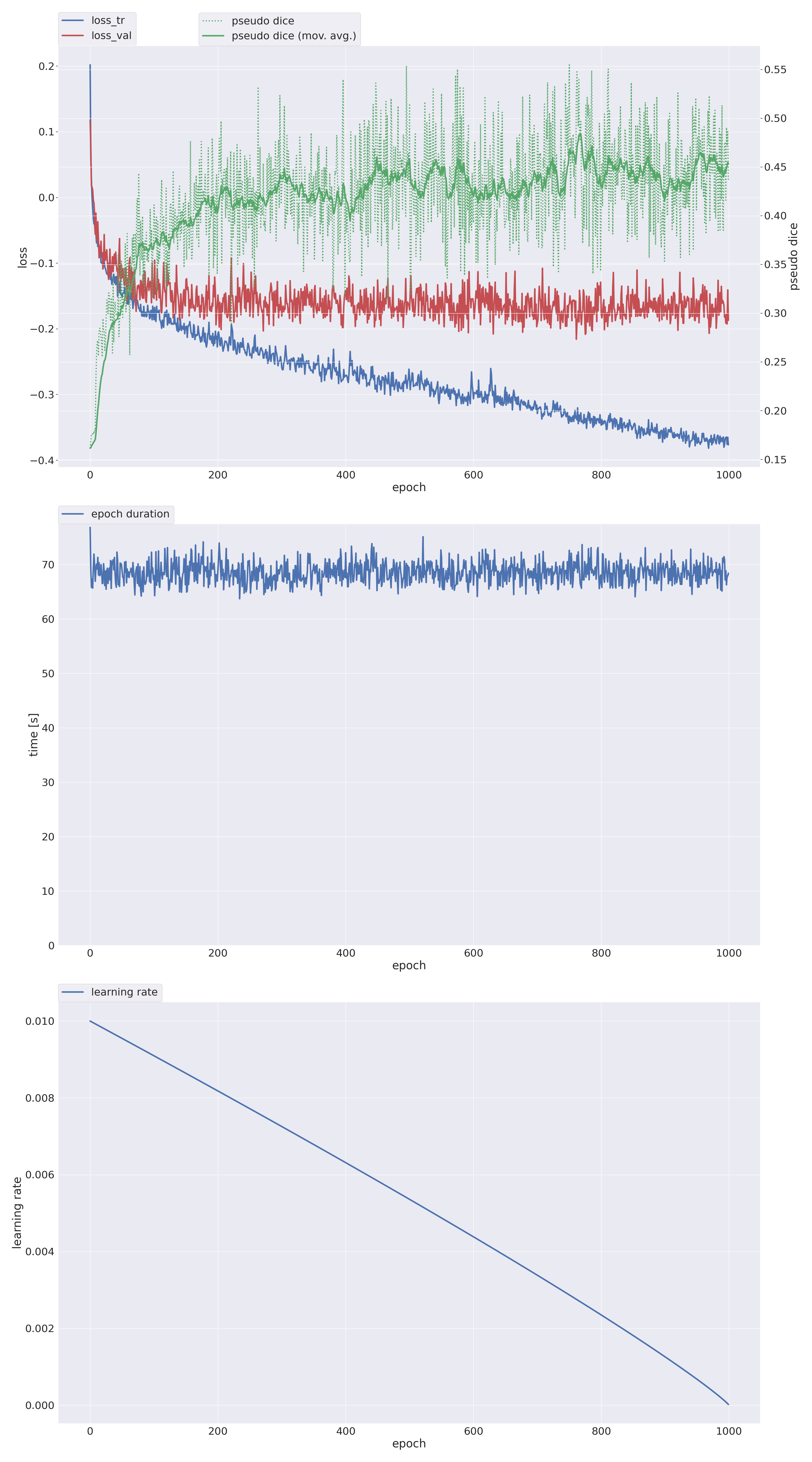

Supplement: Supplementary file 7 — Additional file 7. Learning curves generated by nnU-Net (3d_lowres, 3d_cascade_fullres). [file 40644_2025_844_MOESM7_ESM.zip › learning_curves_2/3d_lowres-fold_1.png]

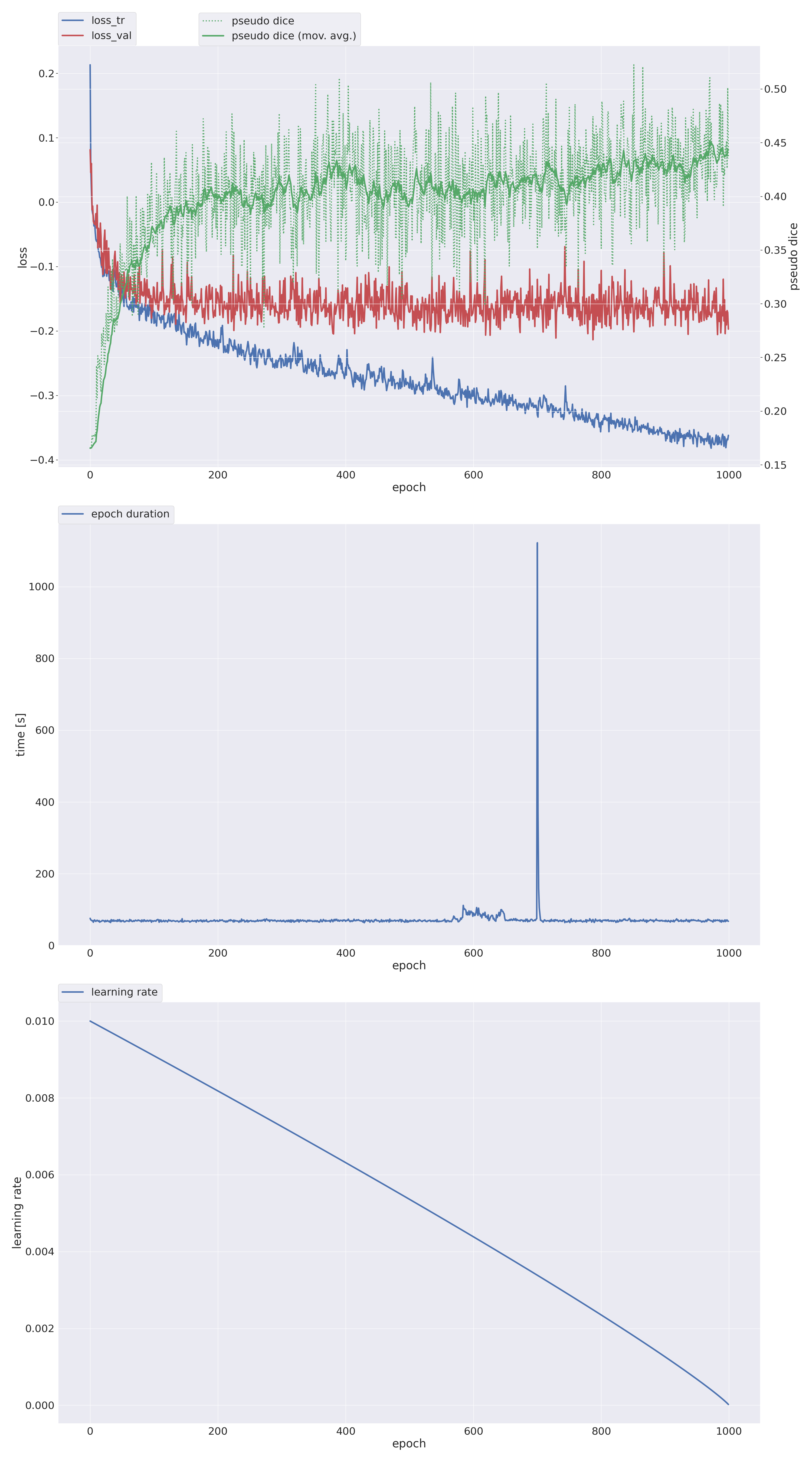

Supplement: Supplementary file 7 — Additional file 7. Learning curves generated by nnU-Net (3d_lowres, 3d_cascade_fullres). [file 40644_2025_844_MOESM7_ESM.zip › learning_curves_2/3d_lowres-fold_2.png]

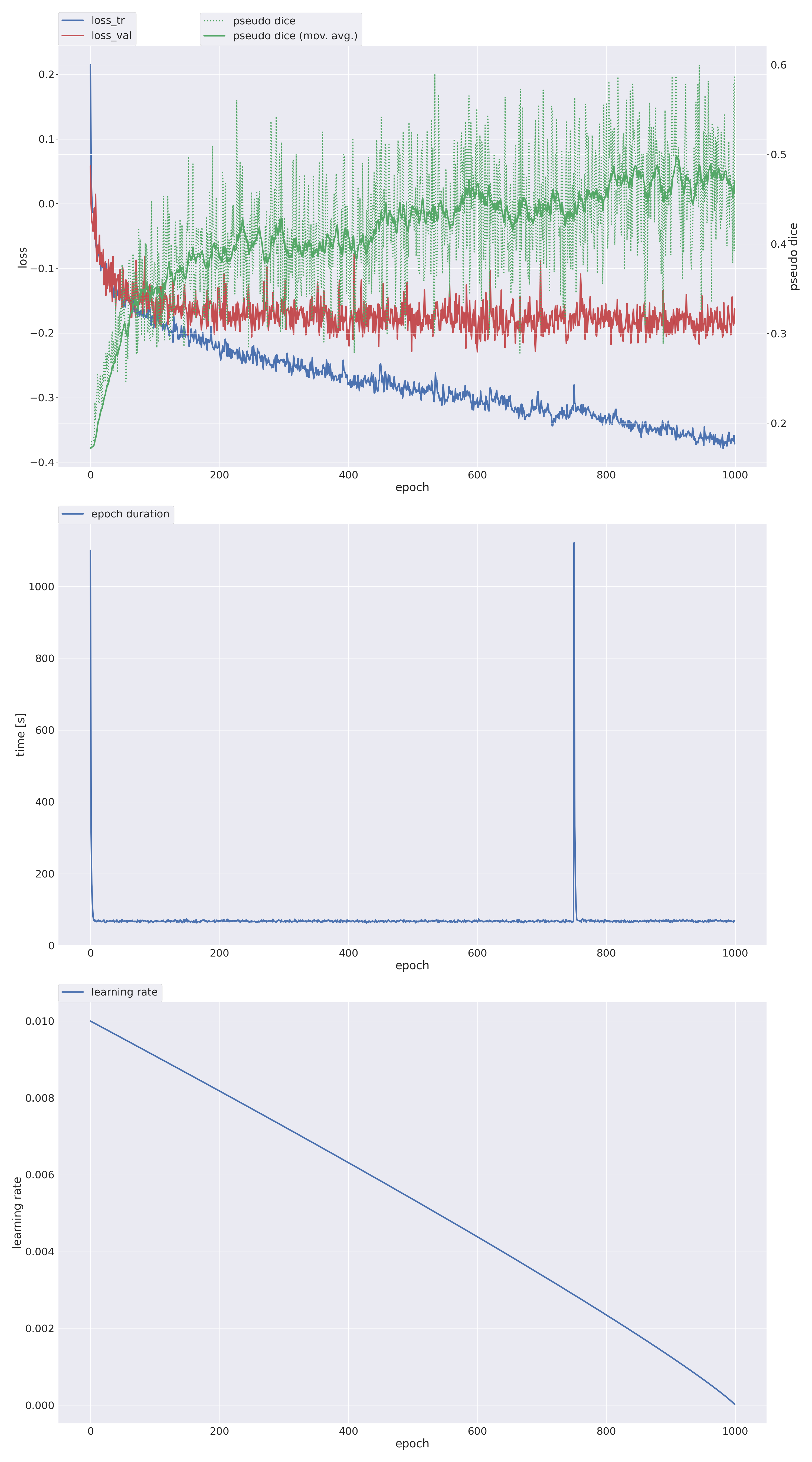

Supplement: Supplementary file 7 — Additional file 7. Learning curves generated by nnU-Net (3d_lowres, 3d_cascade_fullres). [file 40644_2025_844_MOESM7_ESM.zip › learning_curves_2/3d_lowres-fold_3.png]

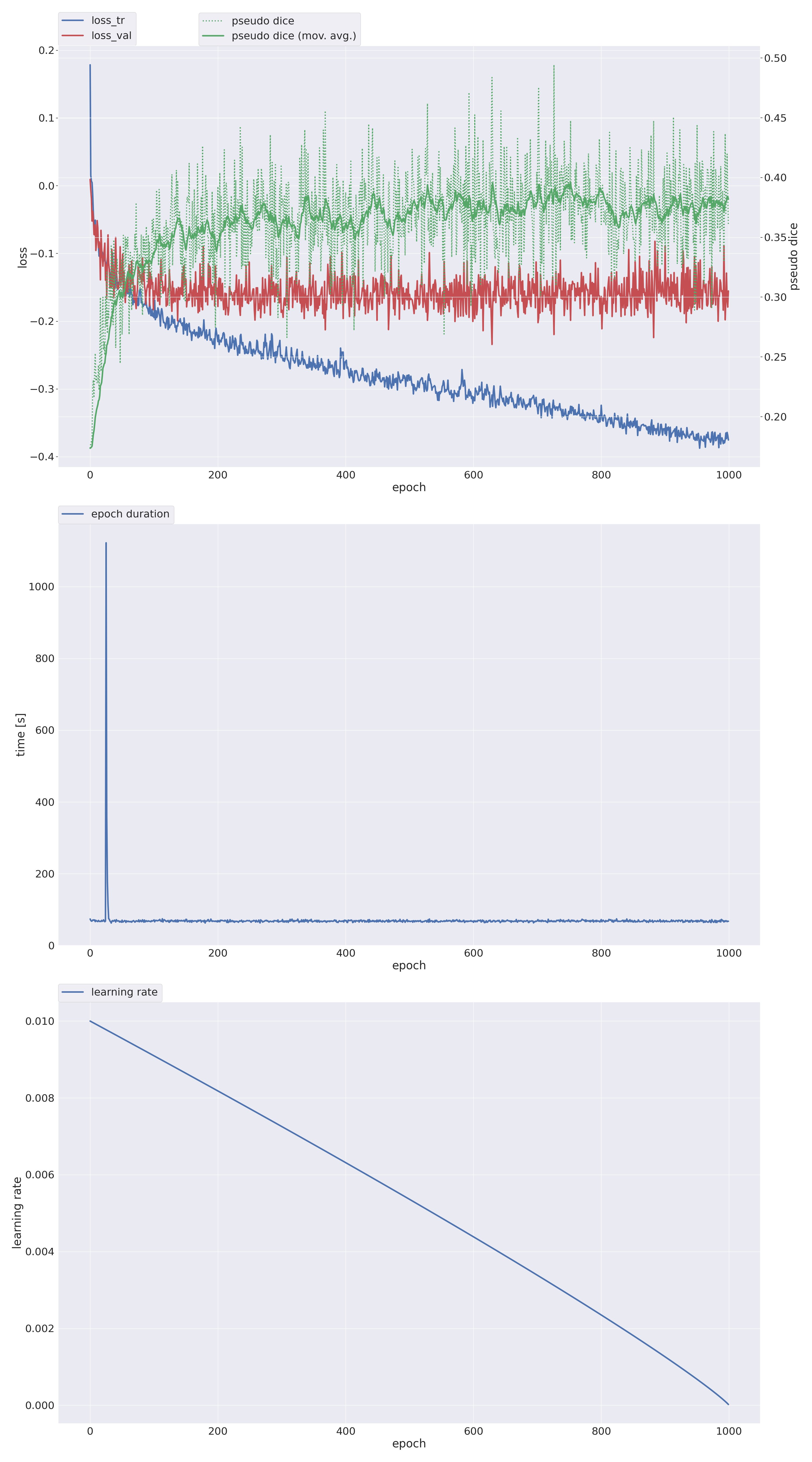

Supplement: Supplementary file 7 — Additional file 7. Learning curves generated by nnU-Net (3d_lowres, 3d_cascade_fullres). [file 40644_2025_844_MOESM7_ESM.zip › learning_curves_2/3d_lowres-fold_4.png]
